# Biochemical and Structural Study of RuvC and YqgF from Deinococcus radiodurans

**DOI:** 10.1128/mbio.01834-22

**Published:** 2022-08-24

**Authors:** Yiyang Sun, Jieyu Yang, Guangzhi Xu, Kaiying Cheng

**Affiliations:** a Department of Immunology and Microbiology, School of Basic Medical Sciences, Hangzhou Normal University, Hangzhou, China; b College of Food and Health, Zhejiang Agriculture and Forestry University, Zhejiang, Lin’an, China; c State Key Laboratory for Diagnosis and Treatment of Infectious Diseases, The First Affiliated Hospital, College of Medicine, Zhejiang University, Hangzhou, China; Duke University School of Medicine

**Keywords:** Holliday junction (HJ), RuvC, RNase, homologous recombination (HR), *Deinococcus*, protein structure

## Abstract

Deinococcus radiodurans possesses robust DNA damage response and repair abilities, and this is mainly due to its efficient homologous recombination repair system, which incorporates an uncharacterized Holliday junction (HJ) resolution process. D. radiodurans encodes two putative HJ resolvase (HJR) homologs: RuvC (DrRuvC) and YqgF (DrYqgF). Here, both DrRuvC and DrYqgF were identified as essential proteins for the survival of D. radiodurans. The crystal structures and the biochemical properties of DrRuvC and DrYqgF were also studied. DrRuvC crystallized as a homodimer, while DrYqgF crystallized as a monomer. DrRuvC could preferentially cleave HJ at the consensus 5′-(G/C)TC↓(G/C)-3′ sequence and could prefer using Mn^2+^ for catalysis *in vitro*, which would be different from the preferences of the other previously characterized RuvCs. On the other hand, DrYqgF was identified as a Mn^2+^-dependent RNA 5′–3′ exo/endonuclease with a sequence preference for poly(A) and without any HJR activity.

## INTRODUCTION

Homologous recombination plays a critical role in generating genetic diversity and repairing DNA lesions, including double-stranded DNA breaks (1). The process occurs in several steps. The initial steps include the introduction of breaks into the DNA and the resection of the DNA into single-stranded DNA tails with 3′ extensions through the combined actions of helicases and nucleases. The intermediate steps include the search for homologous template DNA, the invasion of the strands, and the formation of the joint molecule mediated by the recombinase. The final steps include the migration and the resolution of the joint molecules.

A four-way junction DNA structure, known as the Holliday junction (HJ), is formed during the migration of the joint molecule (1). At the HJ, two homologous duplex DNA molecules are linked by crossovers as a result of strand exchange. HJ can also be formed during replication fork reversal, a process that occurs to rescue stalled forks during DNA replication (2). The resolution of HJs is mediated by HJ resolvases (HJRs), which are a diverse group of DNA structure-specific endonucleases that cleave the two crossover strands across the junction point (3, 4). HJRs have been identified in a wide variety of organisms based on their shared functional characteristics (5–7).

Bioinformatic analyses of evolutionary relationships among HJRs indicated that the independent origin of their functional domain was from four well-defined structural folds, namely, RNase H-like, RusA, endonuclease, and endonuclease VII-colicin E (8). In bacteria, the most well-studied HJR is RuvC, a member of the RNase H-like subfamily (8, 9). It is a dimeric enzyme that resolves HJs by introducing two symmetric 5′-phosphorylated cuts near the center of the HJ (10–13). The Escherichia coli and Helicobacter pylori *ruvC* knockout strain is viable but shows hampered DNA repair efficiency (14, 15). The biochemical studies of E. coli RuvC (EcRuvC), Thermus thermophilus RuvC (TtRuvC), and Pseudomonas aeruginosa RuvC (PaRuvC) indicated that RuvCs function as homodimers, display HJ-specific endonuclease activity, and show sequence preference (13, 16–19). The apo crystal structures of EcRuvC, PaRuvC, and TtRuvC (16–18), as well as the structure of TtRuvC complexed with HJ (20, 21), have already been solved.

The YqgF protein, another member from the RNase H-like subfamily, shares structural similarities with RuvC, is highly conserved among bacterial genomes (8), and also appears in eukaryotic genomes (22). YqgF is essential for the growth of many bacteria, including E. coli (23), Mycobacterium tuberculosis (24), and Salmonella enterica serovar Typhimurium (25). The *yqgF* gene could be successfully deleted in H. pylori and Acinetobacter baylyi ADP1, and both of the knockout strains show impaired growth (26, 27). Unlike the RuvCs, E. coli YqgF exists as a monomer in solution (28). It was reported that EcYqgF lacks HJR activity but is involved in anti-termination at Rho-dependent terminators (29) and in 16S rRNA processing (26, 30). However, in another study, EcYqgF was reported to exhibit nuclease activity on HJs (31). Additionally, EcYqgF was identified as able to effectively cleave both ssRNA and RNA/DNA hybrids as well as physically interact with DNA repair-related proteins and transcription termination factors; therefore, EcYqgF might participate in transcription-coupled DNA repair (31). Furthermore, YqgF homologs, HpDprB and MtRuvX in H. pylori and M. tuberculosis, respectively, have exhibited HJ binding and resolution activity *in vitro* and could promote DNA repair *in vivo* via the dimerization of the monomeric YqgF nuclease domain (27, 32). In a recent study, M. tuberculosis YqgF (MtYqgF, also called MtRuvX) was shown to be capable of hydrolyzing ATP, and the residues essential for ATP binding and for the coordination of Mg^2+^ ions were predicted according to its apo structural features (33). MtYqgF was found to be a nonsequence specific endonuclease that can digest a variety of branched DNA/RNA substrates in the presence of ATP (33). The above-mentioned findings indicate that YqgF may fulfill an important role in the processing of branched DNA recombination intermediates in addition to its essential functions in RNA metabolism.

Deinococcus radiodurans is one of the most radioresistant bacteria, has an efficient homologous recombination repair system, and thus possesses robust DNA damage response and repair abilities (34, 35). However, the late steps of homologous recombination, especially the HJ resolution process in D. radiodurans, are not well-characterized. The D. radiodurans genome possesses homologs of the *ruvC* (*dr0440*) and *yqgf*-like (*dr2509*) genes that encode putative HJRs. Nevertheless, their biological functions and enzymatic properties have not been experimentally assessed. Herein, we report that both the *ruvC* and *yqgF*-like genes are essential for the survival of D. radiodurans. The apo structures of RuvC and YqgF from D. radiodurans (DrRuvC and DrYqgF, respectively) were determined by X-ray crystallography. Furthermore, we found that DrRuvC exhibits HJR activity with a strong preference for Mn^2+^. The cleavage site by DrRuvC occurs preferentially at the 5′-(G/C)TC↓(G/C)-3′ consensus sequence. It was also found that DrYqgF possesses Mn^2+^ dependent RNA 5′–3′ exo/endonuclease activity with a sequence preference for poly(A). To sum up, our study showed that DrRucC might act as a major HJR in D. radiodurans, while DrYqgF might play certain roles in RNA metabolism.

## RESULTS

### Sequence alignments.

**FIG 1:**
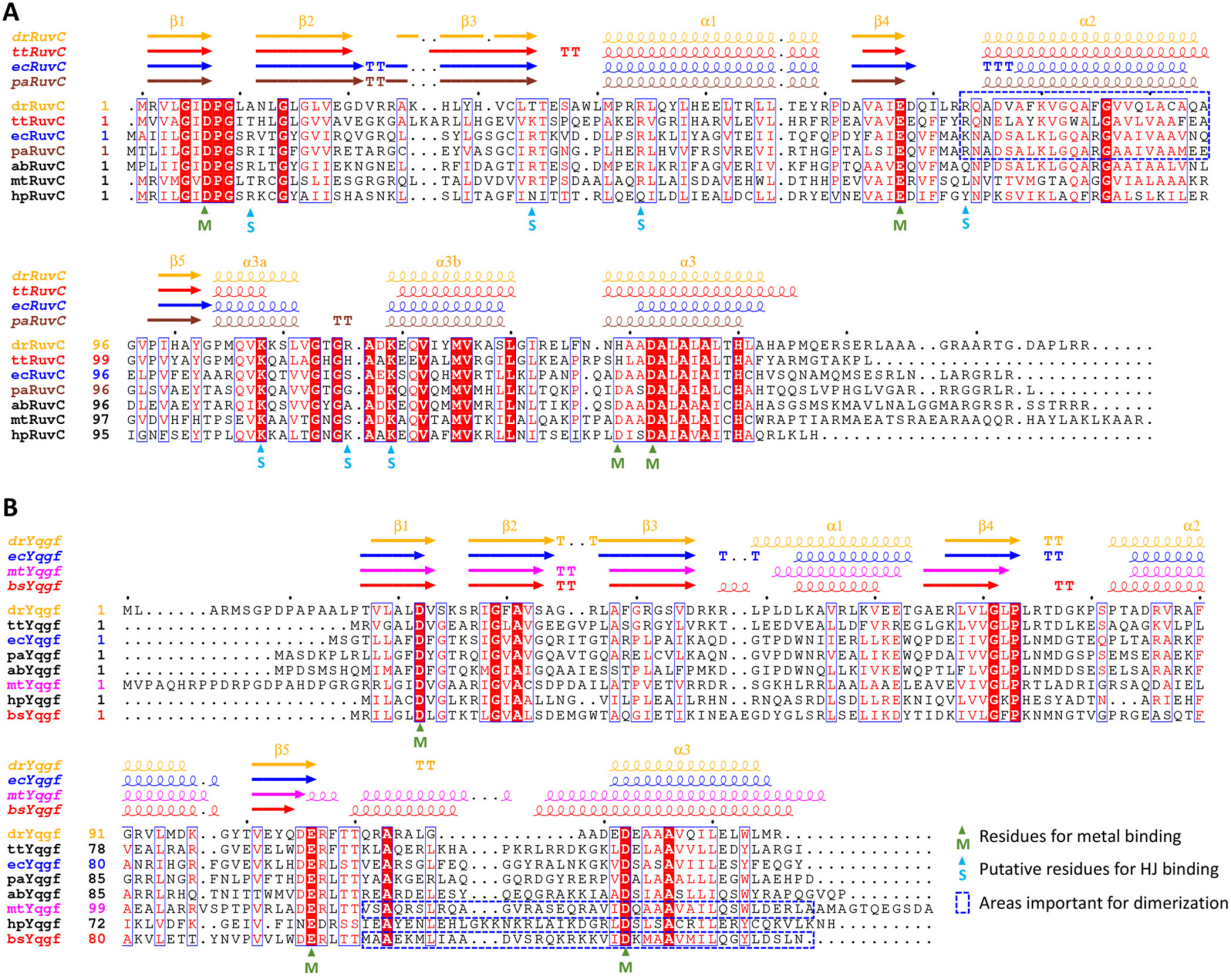
The sequence alignments of RuvC and YqgF. (A) The sequence alignments of RuvC from different organisms. (B) The sequence alignments of YqgF from different organisms. Names of species are dr, Deinococcus radiodurans; tt, Thermus thermophilus HB8; ec, Escherichia coli; pa, Pseudomonas aeruginosa; ab, Acinetobacter baylyi; mt; Mycobacterium tuberculosis; hp, Helicobacter pylori; bs, Bacillus subtilis. Secondary structural elements are depicted according to PDB files (DrRuvC, this study; TtRuvC, PDB code: 4ep4; EcRuvC, PDB code: 1hjr; PaRuvC, PDB code: 6lw3; DrYqgF, this study; EcYqgF, PDB code: 1nu0; MtYqgF, PDB code: 7ess; BsYqgF, PDB code: 1vhx) and are displayed at the top of the sequences. Similar residues are boxed in blue. Conserved key residues are written with white bold characters and are highlighted with a red background. Residues for metal binding and substrate binding are labeled at the bottom of the sequences with green and blue triangles, respectively. Key residues for protein dimerization are framed with a blue dashed box.

**FIG 2:**
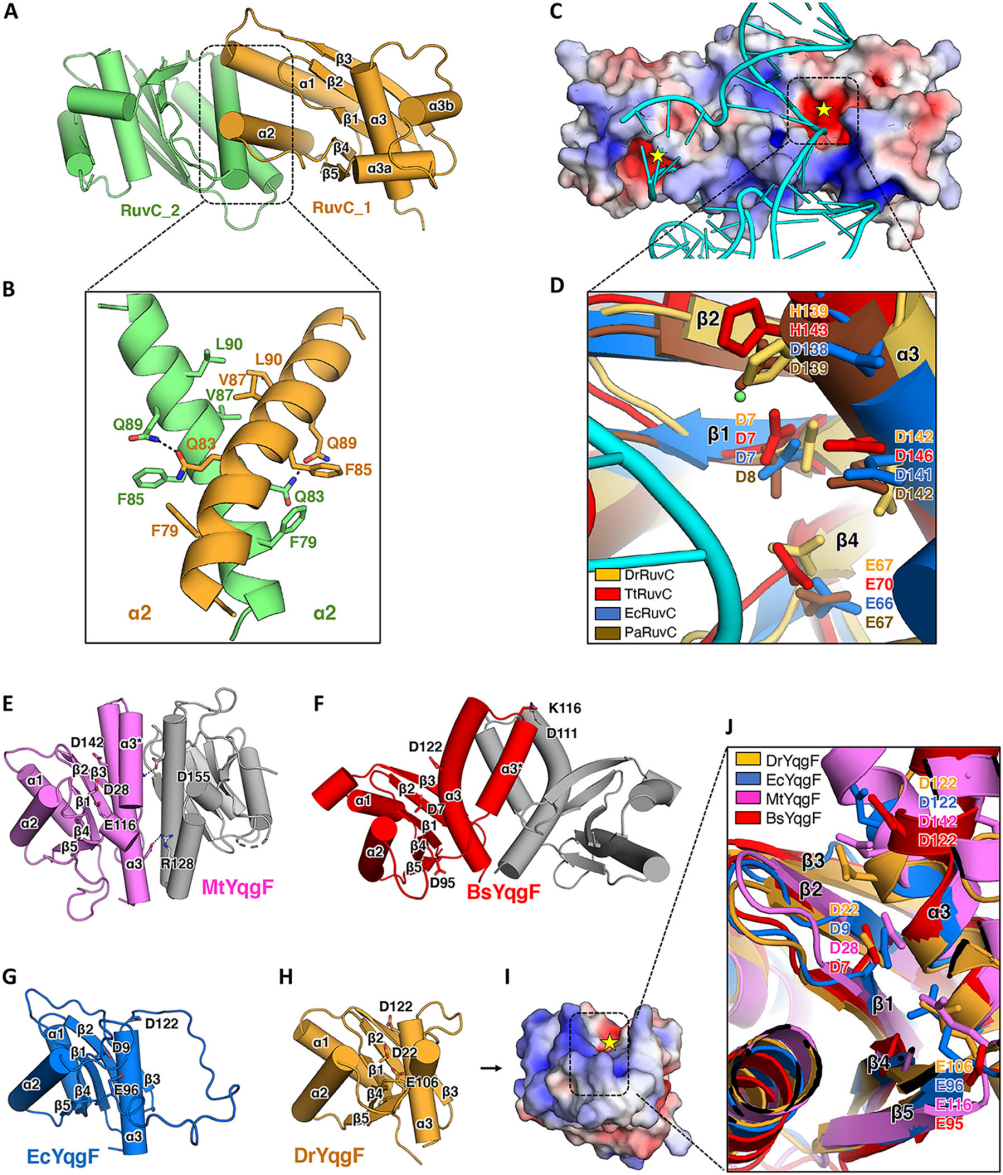
Structure analysis of DrRuvC and DrYqgF. (A) The overall structure of the DrRuvC dimer (this study). The structure elements are numbered and labeled. (B) The zoomed-in view of α2 of DrRuvC (the dimerization area). The key residues for dimerization are labeled and shown as sticks. (C) The electrostatic potential of the DrRuvC surface. The potential was determined using the Adaptive Poisson-Boltzmann Solver (APBS) and is shown as a solvent excluded surface (range = ±5) by PyMOL. The catalytic centers are marked with yellow stars. The HJ substrate, which was extracted from the TtRuvC-HJ complex (PDB code: 6s16), was docked into the DrRuvC apo structure by PyMOL, shown as a cartoon, colored cyan. (D) The zoomed-in view of the aligned catalytic center of DrRuvC (orange; this study), TtRuvC (red; PDB code: 4ep4), EcRuvC (blue; PDB code: 1nmn), and PaRuvC (brown; PDB code: 6lw3). The key putative catalytic residues are labeled and shown as sticks. (E) The overall structure of the MtYqgF dimer (PDB code: 7ess). Each monomer is colored differently. The structure elements are numbered and labeled. The putative catalytic residues and the key residues for dimerization are labeled and shown as sticks. (F) The overall structure of the BsYqgF dimer (PDB code: 1vhx). Each monomer is colored differently. The structure elements are numbered and labeled. The putative catalytic residues and key residues for dimerization are labeled and shown as sticks. (G) The overall structure of EcYqgF (PDB code: 1ovq). The structure elements are numbered and labeled. The putative catalytic residues are labeled and shown as sticks. (H) The overall structure of DrYqgF (this study). The structure elements are numbered and labeled. The putative catalytic residues are labeled and shown as sticks. (I) The electrostatic potential of the DrYqgF surface. The potential was determined using the APBS and is shown as a solvent excluded surface (range = ±5) by PyMOL. The catalytic center is marked with a yellow star. (J) The zoomed-in view of the aligned catalytic center of DrYqgF (orange; this study), EcYqgF (blue; PDB code: 1ovq), MtYqgF (violet; PDB code: 7ess), and BsYqgF (red; PDB code: 1vhx). The key putative catalytic residues are labeled and shown as sticks.

The widespread bacterial RuvC and YqgF are evolutionarily connected, and both possess a canonical RNase H-like fold, including five β-strands (β1 to β5) and three α-helices (α1 to α3) (Fig. 1). RuvC is a relatively conserved protein. The percentage of sequence identity between DrRuvC and RuvC from other bacteria (such as T. thermophilus HB8, E. coli, P. aeruginosa, A. baylyi, M. tuberculosis, and H. pylori) is >30% (Table S2A). Both a sequence alignment and a structure comparison of RuvC showed three conserved acidic catalytic residues (located at β1, β4, and α3) that constitute its catalytic center (Fig. 1A and Fig. 2D) (36). The fourth catalytic residue of RuvC, which is also located at α3 and is usually glutamate, is replaced by histidine in DrRuvC and TtRuvC (Fig. 1A). RuvC contains two extra α-helices (α3a and α3b) between β5 and α3, which are essential for the binding of HJ (20, 21, 37). These two extra α-helices make the length of the universal RuvC greater than that of the YqgF. With limited biochemical data and a lack of substrate-bound complex structures, the substrate binding sites on YqgF have not yet been identified. Compared with RuvC, YqgF is a less conserved protein. Although it share high identities (>29%) with TtYqgF, EcYqgF, and PaYqgF, DrYqgF only shares 25%, 20%, and 29% identity with AbYqgF, HpYqgF, and MtYqgF, respectively (Table S2B). Both HpYqgF and MtYqgF exhibit HJ resolvase activities. The locations of the catalytic residues of RuvC and YqgF are different. The conserved glutamate at the C terminus of β4 (Fig. 1A) on RuvC is missing in YqgF proteins. Instead, YqgF contains conserved glutamate at the C terminus of β5 (Fig. 1B). Furthermore, there are two putative catalytic residues on α3 in RuvC, but there is only one in YqgF (Fig. 1A and B). Canonical RNase H-like enzymes contain four conserved carboxylates in their active sites, allowing for the positioning of two catalytic metal ions (38). There are only three carboxylates on YqgFs for metal-chelating, which is not enough to chelate two metal ions. It has been hypothesized that the RNase H-like domain of Prp8 may form a composite nuclease active site together with the functional groups from the bound RNA substrate (39). Therefore, the presence of a substrate might also help YqgFs to form the active site.

10.1128/mbio.01834-22.3 TABLE S2 The percentages of amino acid sequence identities. (A) The percentages of amino acid sequence identities between the RuvC resolvases. (B) The percentages of amino acid sequence identities between the YqgFs. Download Table S2, PDF file, 0.1 MB. Copyright © 2022 Sun et al. 2022 Sun et al. https://creativecommons.org/licenses/by/4.0/ This content is distributed under the terms of the Creative Commons Attribution 4.0 International license.

### Disruption of *drruvC* and *dryqgF*.

A universally acknowledged gene knockout technique for D. radiodurans (see Materials and Methods), based on the intrinsic efficient homologous recombination of this strain (40, 41), was applied to disrupt the *drruvC* and *dryqgF* genes in this study. However, we only obtained heterozygotes for *drruvC* and *dryqgF* after several rounds of screening (Fig. S1), indicating that *drruvC* and *dryqgF* could be essential genes for D. radiodurans.

10.1128/mbio.01834-22.4 FIG S1 Construction and verification of the deletion mutants. Upper, schematic of the constructions of the *drruvC* (A) and *dryqgF* (B) deletion mutants. The maps of *drruvC* (A) and *dryqgF* (B) in the D. radiodurans chromosome before (top) or after (bottom) replacement with a kanamycin resistance cassette are shown. Lower, ethidium bromide-stained agarose gel illustrating that the mutants carry heterozygous deletions of *drruvC* (A) or *dryqgF* (B). WT, wild type strain; m1 to m10, different mutant strains. Download FIG S1, PDF file, 0.3 MB. Copyright © 2022 Sun et al. 2022 Sun et al. https://creativecommons.org/licenses/by/4.0/ This content is distributed under the terms of the Creative Commons Attribution 4.0 International license.

### Crystal structures of DrRuvC and DrYqgF.

Despite designing many different substrates for DrRuvC and DrYqgF, and despite the complex structure screenings being conducted via trial-and-error, only the apo structures of both proteins could be obtained. The crystallographic statistics are presented in Table 1.

**TABLE 1:** Statistics from crystallographic analysis

Complex	DrRuvC	DrYqgF
PDB code	7W8D	7W89
Data collection		
Source	BL02U1	BL02U1
Wavelength (Å)	0.9792	0.9792
Resolution (Å)	27.71-2.75 (2.79 to 2.75)*	27.07-1.5 (1.54 to 1.5)^*a*^
Space group	P212121	C121
Cell dimensions: a, b, c	41.20, 72.77, 112.3	73.07, 49.38, 33.52
Obeservation	48924 (3546)^*a*^	96829 (7255)^*a*^
Unique reflections	9594 (674)^*a*^	19580 (1430)^*a*^
R_merge_ (%)	13.1 (72.2)^*a*^	5.5 (13.2)*
I/σI	13.4 (2.56)^*a*^	26.04 (15.20)^*a*^
Completeness (%)	91.2 (93.4)^*a*^	93.7 (95.4)^*a*^
Redundancy	4.0	4.5
Refinement statistics		
R_factor_^*b*^ (%)/R_free_^*c*^ (%)	27.34/30.58	19.48/19.56
rmsd bonds (Å)/angles (°)	0.004/0.757	0.01/1.043
Ramachandran plot: Favored (%)	95.6	98.4

*^a^*The numbers in parentheses refer to the last shell.

*^b^*R_factor_ = Σ‖F(obs) − F(calc)‖ / Σ|F(obs)|.

*^c^*R_free_ = R factor calculated using 5.0% of the reflection data. These data were randomly chosen and were omitted from the start of the refinement.

The crystal structure of DrRuvC was determined by molecular replacement, using the structure of TtRuvC (PDB code: 4ep4) as the search model. The final structure was refined to a resolution of 2.75 Å, and its R_factor_ and R_free_ values were estimated to be 27.34% and 30.58%, respectively. The refined DrRuvC model contains two monomer molecules in an asymmetric unit. Each monomer is comprised of five β-strands (β1 to β5), and these strands are sandwiched by five α-helices (α1, α2, α3a, α3b, and α3), forming a canonical Rossman fold (Fig. 2A). The DrRuvC dimer structure superimposed well with the TtRuvC structure (the root-mean-square deviation [RMSD] value for 258 Cα atoms is 1.665 Å), EcRuvC structure (the RMSD value for 268 Cα atoms is 1.933 Å), and PaRuvC structure (the RMSD value for 280 Cα atoms is 2.111 Å) (Fig. S2B), in agreement with their high conservations of amino acid sequences (Fig. 1A; Table S2A). The two monomers of DrRuvC are bound together through interactions, mainly between α2, involving both polar (Q83 and Q89) and hydrophobic interactions (F79, F85, V87, and L90) (Fig. 2B). Interestingly, the DrRuvC homodimer exhibits distinct asymmetry in residues 73 to 83 spanning the dimer interface, which is also observed in the TtRuvC apo structure (Fig. S2A). However, such asymmetry is not present in the apo structures of EcRuvC (PDB code: 1hjr), PaRuvC (PDB code: 6lw3), or the complex structure of TtRuvC-HJ (PDB code: 6s16). The high-temperature structure factor values indicate that the area around the DrRuvC residues 73 to 83 is not rigid (Fig. S2D) and might undergo a disorder-to-order transition upon HJ substrate binding. Although the crystal condition contains 0.2 M MgCl_2_, only weak metal ion density was observed in the DrRuvC structure. A putative HJ substrate could be docked into a DrRuvC dimer, according to the superimposition of the DrRuvC structure and the TtRuvC-HJ complex structure (PDB code: 6s16) (Fig. 2C). The active sites of DrRuvC were predicted by 2D and 3D alignments. These putative catalytic residues of DrRuvC are conserved with those of TtRuvC. However, H139 is replaced by D138 in EcRuvC and D139 in PaRuvC (Fig. 2D).

10.1128/mbio.01834-22.5 FIG S2 Structural analysis of RuvCs and YqgFs. (A) Superimpositions of the two molecules from the DrRuvC apo homodimer structure (this study), TtRuvC apo homodimer structure (PDB code: 4ep4), EcRuvC homodimer structure (PDB code: 1hjr), PaRuvC homodimer structure (PDB code: 6lw3), and TtRuvC homodimer–HJ complex structure (PDB code: 6s16). Each molecule of the dimer is shown in a different color. (B) Superimpositions of the overall structures of the RuvC dimers. DrRuvC (orange; this study), TtRuvC (red; PDB code: 4ep4), EcRuvC (blue; PDB code: 1nmn), and PaRuvC (brown; PDB code: 6lw3) were shown as cartoons and superimposed. (C) Superimposition of the overall structures of YqgF. DrYqgF (orange; this study), EcYqgF (blue; PDB code: 1ovq), MtYqgF (violet; PDB code: 7ess), and BsYqgF (red; PDB code: 1vhx) were shown as cartoons and superimposed. (D) The temperature factor of the DrRuvC dimer. The temperature factor was calculated by PyMOL, and the structure was shown as cartoon putty and colored according to the spectrum of the temperature factor values. The higher the factor value, the bigger the size of the cartoon putty and the warmer the color. The direction of view is the same as that of (B). (E) The temperature factor of DrYqgF. All of the settings are the same as those of (D), and the direction of the view is the same as that of (C). Download FIG S2, PDF file, 0.9 MB. Copyright © 2022 Sun et al. 2022 Sun et al. https://creativecommons.org/licenses/by/4.0/ This content is distributed under the terms of the Creative Commons Attribution 4.0 International license.

The crystal structure of DrYqgF was determined by molecular replacement, using the structure of EcYqgF (PDB code: 1ovq) as the search model. The final structure was refined to a resolution of 1.5 Å, and its R_factor_ and R_free_ values were determined to be 19.48% and 19.56%, respectively. The refined model of DrYqgF contains one DrYqgF molecule in an asymmetric unit, and each monomer is comprised of five β-strands (β1 to β5), and these strands are sandwiched by three α-helices (α1, α2, and α3) (Fig. 2H). Besides the significantly flexible residues between β5 and α3, the structure of DrYqgF superimposes well with that of EcYqgF (the RMSD value for 82 Cα atoms is 3.184 Å), MtYqgF (PDB code: 7ess; the RMSD value for 95 Cα atoms is 2.335 Å), and Bacillus subtilis YqgF (PDB code: 1vhx; the RMSD value for 96 Cα atoms is 2.876 Å) (Fig. S2C). The current putative active site on the DrYqgF structure contains an incomplete set of metal-chelating residues, which is insufficient to chelate two metal ions. However, the presence of a substrate may provide functional groups with which to form a composite nuclease active site. Furthermore, the current putative active site of DrYqgF is too narrow for metal ions or for substrate binding. The presence of a substrate might also assist DrYqgF in undergoing a conformational change, thereby enabling the occupation of metal ions and substrate.

The topologies of RuvC and YqgF are quite similar, except the α3a and α3b areas, which might be important for HJ binding in RuvC, is not present in YqgF. The residues between β5 and α3 in YqgF are not conserved and are too short to form two long α-helices as in RuvC. The area between β5 and α3 is disordered in the EcYqgF crystal structure (PDB code: 1nmn). The NMR structure of EcYqgF (PDB code: 1ovq) indicated that this area is a flexible loop that has multiple states (Fig. 2G) (28). However, in the crystal structure of MtYqgF, the area between β5 and α3 (residues 120 to 131) forms a long α-helix (α3*). From the MtYqgF dimer structure, it seems that two unconserved polar residues on α3* (R128) and α3 (D155) are essential for dimerization (Fig. 2E) (33). BsYqgF also crystallizes as dimers, and such dimerization is mediated by the residues on α3* and α3, as well (for example, polar interactions between D111 and K116; PDB code: 1vhx) (Fig. 2F). In the DrYqgF crystal structure, the residues between β5 and α3, especially residues 108 to 111, tend to form a short α-helix, although the remaining residues are also flexible (Fig. 2H). DrYqgF does not possess residues that correspond to MtYqgF R128 and D155 or to BsYqgF D111 and K116, which is important for dimerization; however, other residues that might also participate in the dimerization cannot be ruled out. The high-temperature structure factor of DrYqgF indicates that the area between β5 and α3 is nonrigid (Fig. S2E). Nevertheless, this area may become ordered upon substrate binding or under specific conditions. Similar to the DrRuvC structure, although the crystallization condition contained 0.2 M MgCl_2_, no obvious metal ion density was observed in the DrYqgF structure (Fig. 2I and J). A possible cause may be that the coordination of metal ions requires the correct catalytic geometry, which requires the appearance of the substrate.

### Dimerization analysis of DrRuvC and DrYqgF.

**FIG 3:**
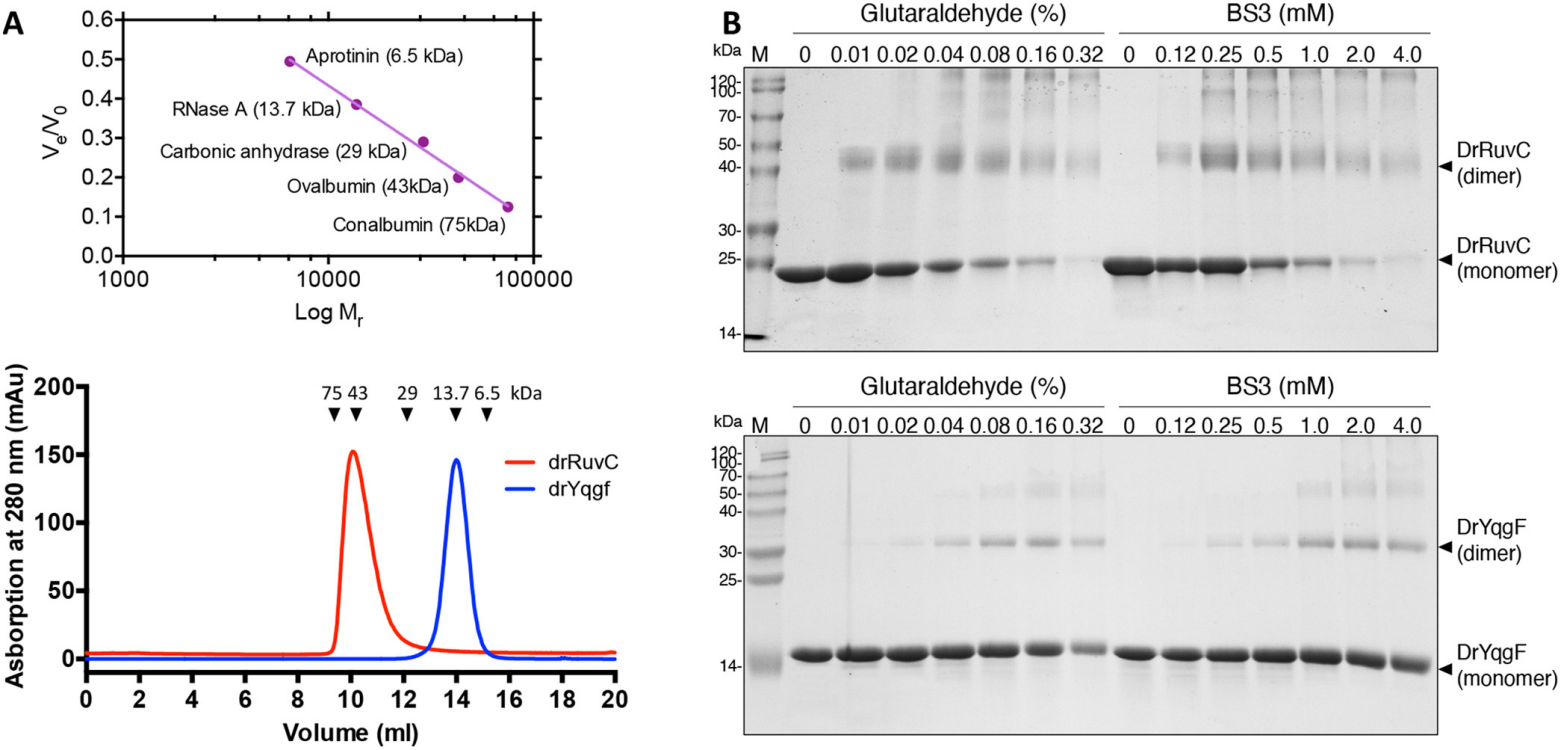
Dimerization analysis of DrRuvC and DrYqgF. (A) The gel filtration analysis of DrRuvC and DrYqgF. A set of protein standards of known molecular mass, such as aprotinin (6.5 kDa), RNase A (13.7 kDa), carbonic anhydrase (29 kDa), ovalbumin (43 kDa), and conalbumin (75 kDa), were used to calibrate the Superdex 75 10/300GL. DrRuvC eluted around 10 mL, reflecting a dimer form. DrYqgF eluted around 14 mL, reflecting a monomer form. (B) The protein cross-linking assays of DrRuvC and DrYqgF. The upper gel showed the cross-linking result of DrRuvC, and the lower gel showed the cross-linking result of DrYqgF. Lanes 1 and 8: DrRuvC or DrYqgF protein without the cross-link reagent treatment. Lanes 2 to 7: proteins were treated with different concentrations of glutaraldehyde (0.01, 0.02, 0.04, 0.08, 0.16, and 0.32%). Lanes 9 to 14, proteins were treated with different concentrations of BS3 (0.125, 0.25, 0. 5, 1, 2, and 4 mM).

Although from structural data, DrRuvC was determined to be a homodimer and DrYqgF was determined to be a monomer, it is not known whether DrRuvC and DrYqgF are functionally active as dimers in solution. To examine this, analytical size exclusion chromatography assays were conducted (Fig. 3A). Both DrRuvC and DrYqgF were eluted in major peaks, except that the eluted peak of DrRuvC corresponded to a molecular weight of approximately 40 kDa, and the eluted peak of DrYqgF corresponded to a molecular weight of approximately 15 kDa. The predicted molecular massed of DrRuvC and DrYqgF were 20 kDa and 15 kDa, respectively. Hence, it seems that DrRuvC exists as a homodimer and that DrYqgF exists as a monomer in solution. However, we did observe the formation of dimer bands of DrYqgF by sodium dodecyl sulfate-polyacrylamide gel electrophoresis (SDS-PAGE) when glutaraldehyde and bis-sulfo-succinimidyl suberate (BS3) were selected as cross-linking reagents to further analyze the dimerization (Fig. 3B). Therefore, we considered that DrYqgF tends to dimerize at specific conditions. Whether the specific conditions for DrYqgF dimerization require higher protein concentrations or the addition of metal ions or substrates still needs to be studied.

### Analysis of Holliday junction resolvase activities of DrRuvC and DrYqgF.

**FIG 4:**
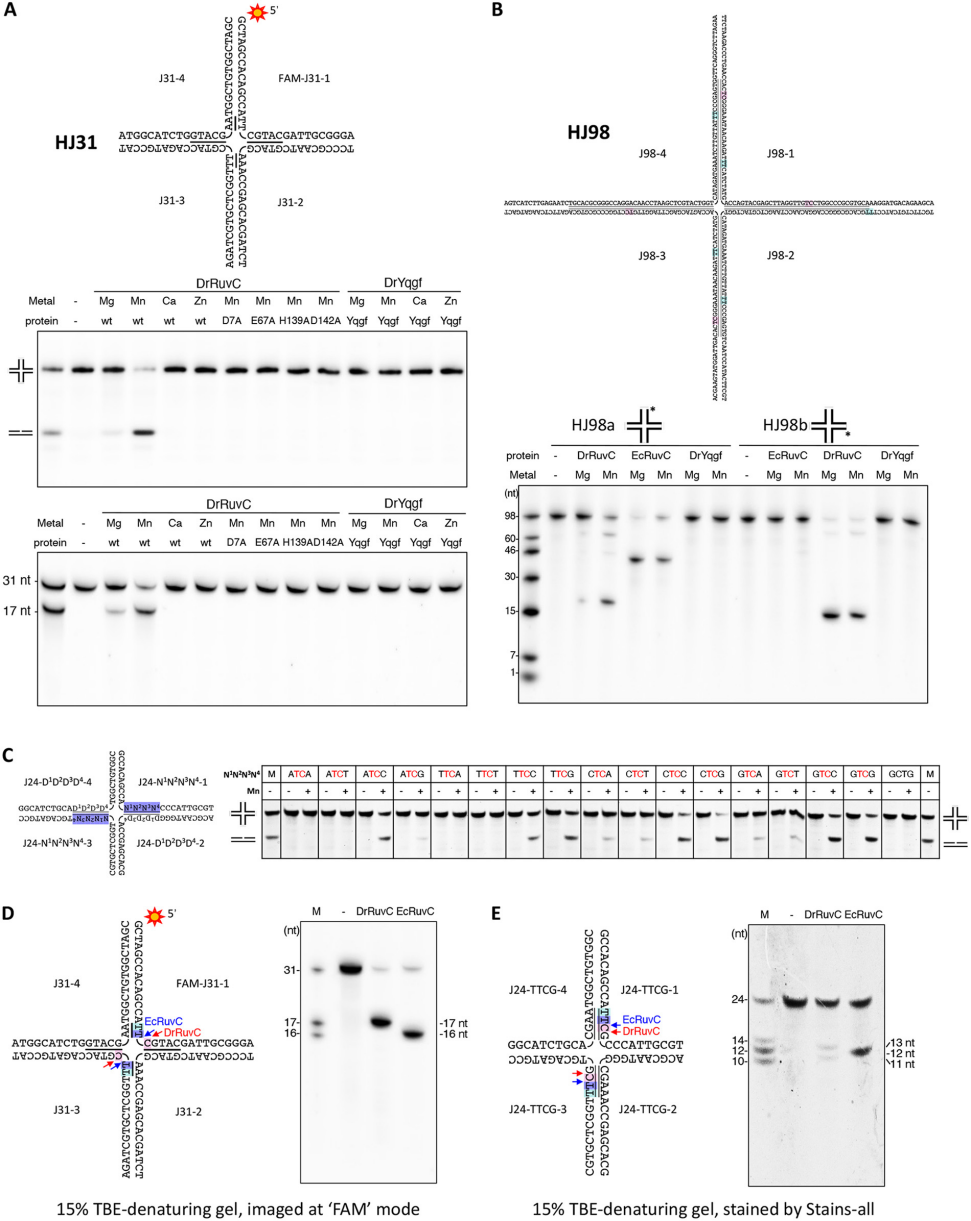
Holliday junction resolvase assays of DrRuvC and DrYqgF. (A) Analysis of the HJR activities of DrRuvC and DrYqgF over short HJ (HJ31) with a short mobile junction. Together with three other unlabeled strands (J31-2, 3 and 4), a 5′ 6-FAM labeled strand (J31-1) which contains the putative RuvC cleavage site (5′-ATTC-3′) at the mobile junction area was annealed into the HJ substrate HJ31. The underlined bases in the HJs correspond to the homologous core. 200 nM DNA was mixed with 1 μM protein, 10 mM metal (Mg^2+^, Mn^2+^, Ca^2+^, or Zn^2+^), and products were resolved by 10% native TBE-PAGE (middle) and 12% TBE-urea denaturing gels (lower) in the same time. (B) Analysis of the HJR activities on long HJ substrates (HJ98) with a long mobile junction. 5′ 6-FAM labeled HJ substrates with a 66 nt homologous core were synthesized (labeled at strand J98-1 or J98-2) to monitor the different cutting patterns by DrRuvC. 200 nM DNA was mixed with 1 μM protein and 10 mM metal (Mg^2+^ or Mn^2+^) and then incubated at 37°C for 30 min. Products were resolved by 15% TBE-urea denaturing gel. (C) Analysis of the preferred cleavage sequences of DrRuvC. Holliday junctions are different only in the homologous core sequence indicated as N^1^N^2^N^3^N^4^. Reactions for each substrate were performed at the same conditions. 2.5 μM DNA was mixed with 5 μM DrRuvC and 10 mM Mn^2+^ and then incubated at 37°C for 30 min. Products were resolved by 10% native TBE-PAGE and stained by Stains-all. (D) and (E) Mapping the cleavage sites of DrRuvC. 5′ 6-FAM labeled HJ31 (D) and unlabeled HJ24-TTCG (E) substrates were used for a cleavage sites analysis, and reactions were performed at the same conditions as in (A) and (C), respectively. The gels were imaged by fluorescence mode or after staining with Stains-all. The cleavage sites of DrRuvC were determined by comparing the position of the product bands with the marker bands and the product bands created by EcRuvC.

It was reported that the HJ cleavage by EcRuvC, TtRuvC, PaRuvC, and HpYqgF (hpDprB) occurs preferentially at the 5′-(A/T)TT↓(G/C)-3′ consensus sequence (17, 18, 21, 27, 36) and that the HJ cleavage by MtYqgF occurs preferentially at the 5′-GT↓CC-3′ consensus sequence (32). To test the HJR activities of DrRuvC and DrYqgF, we first designed a short HJ substrate (HJ31) with the 6-FAM-labeled strand containing a 5′-TTCGTAC-3′ cognate sequence at the mobile junction area for an HJR activity study. It was found that the wild type DrRuvC could resolve HJ31 by introducing two symmetric cuts on the cognate sequence (Fig. 4A). Replacement by alanine of the putative catalytic residues in DrRuvC (D7A, E67A, H139A, and D142A) abolished its HJR activity (Fig. 4A).

A longer HJ substrate (HJ98) which contained a 66 nt cognate sequence at the mobile junction area was synthesized for further HJR activity analysis of DrRuvC and DrYqgF (Fig. 4B). HJ98 contains the cleavage site of EcRuvC, 5′-TTTC-3′, and the cleavage site of MtYqgF, 5′-GTCC-3′. EcRuvC was set as a control in the same assay. Both DrRuvC and EcRuvC exhibited HJR activity over this substrate. However, DrRuvC displayed different cutting patterns than did EcRuvC (Fig. 4B). Different cutting patterns were also detected when another HJ substrate (HJ60) was used (Fig. S3). No HJR activity was detected for DrYqgF, irrespective of the different HJ substrates (HJ31, HJ98, or HJ60) being used (Fig. 4A and B; Fig. S3).

10.1128/mbio.01834-22.6 FIG S3 Analysis of the HJR activities of DrRuvC and DrYqgF on long HJ substrates (HJ60). 5′ 6-FAM labeled HJ substrates with a 22 nt homologous core were synthesized (labeled at strand J60-1 or J60-2) to monitor the different cutting patterns. 200 nM DNA was mixed with 1 μM protein and 10 mM metal (Mg^2+^ or Mn^2+^) and then incubated at 37°C for 30 min. Products were resolved by 15% TBE-urea denaturing gel. EcRuvC was set as a control. Download FIG S3, PDF file, 0.2 MB. Copyright © 2022 Sun et al. 2022 Sun et al. https://creativecommons.org/licenses/by/4.0/ This content is distributed under the terms of the Creative Commons Attribution 4.0 International license.

According to the positions of DNA marker bands and the product bands created by EcRuvC, it seems that DrRuvC prefers to introduce a cut at the 5′-TC-3′ consensus sequence. However, DrRuvC has a preference for the specific bases at the ends of 5′-TC-3′, as not all 5′-TC-3′ sequences would be cut (Fig. 4B; Fig. S3). Therefore, we set up assays to analyze the sequence-specificity of DrRuvC over a series of synthetic HJs which contained a 4 nt cognate sequence at the mobile junction area. These HJs differed at the 5′ end or the 3′ end of the 5′-TC-3′ consensus sequence. It was found that DrRuvC prefers to cut 5′-TC-3′ when a guanosine (G) or cytosine (C) base appeared at the ends (Fig. 4C). However, the activity would be weakened or inhibited if an adenine (A) or thymine (T) base appeared at the ends of 5′-TC-3′ (Fig. 4C).

To identify the exact cleavage site of DrRuvC, two HJ substrates, both of which contained 5′-TTCG-3′, were used for further analysis. EcRuvC was set as a control. As expected, both HJ substrates can be resolved by DrRuvC and EcRuvC, but the cutting patterns are different. It was found that DrRuvC cleaves the HJ after a cytosine residue, different from EcRuvC, which cleaves the HJ after a thymine residue (Fig. 4D and E). To sum up, the HJ cleavage by DrRuvC occurs preferentially at the 5′-(G/C)TC↓(G/C)-3′ consensus sequence.

**FIG 5:**
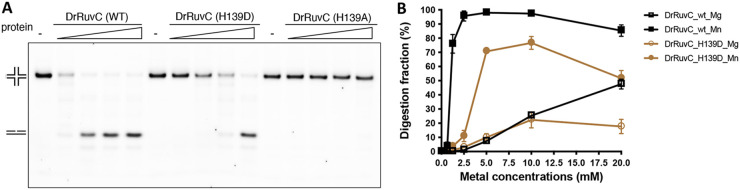
Catalytic metal concentrations analysis of DrRuvC. (A) Comparison of the HJR efficiencies of wild type DrRuvC, H139D mutant, and H139A mutant. Different concentrations of protein (0.5, 1, 2, and 4 μM) were incubated with 200 nM HJ31 and 10 mM Mn^2+^, and the reaction products were resolved by 10% native TBE-PAGE. (B) Analysis of the metal preference of wild type DrRuvC and H139D mutant. Different concentrations (0.31, 0.625, 1.25, 2.5, 5, 10, and 20 mM) of Mg^2+^ or Mn^2+^ were added into the reaction system, and the products were resolved by 8% native TBE-PAGE (see Fig. S4A for one of the representative gel results). The digestion fractions were calculated by Image J from three repeats and displayed as a line chart using GraphPad Prism 9.

Moreover, DrRuvC prefers using Mn^2+^ as a cofactor for catalysis (Fig. 4A and B; Fig. S3), and the optimum concentration of Mn^2+^ is 2.5 to 10 mM (Fig. 5B; Fig. S4A). No HJR activity was detected in the presence of Ca^2+^ or Zn^2+^. However, EcRuvC and PaRuvC prefer using Mg^2+^ for catalysis (17, 36). Unlike the RuvCs from the *Deinococcus-Thermus* phylum, the corresponding residue of H139 is an aspartic acid in other bacteria (Fig. 1A and 2D). We wonder whether the evolutionary replacement of aspartic acid with histidine affects the HJR activity and catalytic metal selection. The H139D mutant of DrRuvC showed reduced activity in the presence of Mn^2+^ and a slightly enhanced activity in the presence of Mg^2+^ over the HJ31 substrate (Fig. 5; Fig. S4A). At the same time, a catalytic metal concentration analysis of EcRuvC was also conducted over the same HJ substrate. Unlike DrRuvC, wild type EcRuvC exhibited slightly enhanced activity when Mg^2+^ took the place of Mn^2+^. Meanwhile, replacement by histidine of D138 on EcRuvC yielded significantly impaired HJR activity. Although the D138H mutant completely lost activity in the presence of Mg^2+^, weak activity was detected when high concentrations of Mn^2+^ appeared. Therefore, it seems that H139 is important for the selection of the metal cofactor for catalysis, even though it influences the HJ resolution efficiency differently among the different RuvCs. Since Mn^2+^ possesses a lower pKa value than does Mg^2+^, the reason for the switching of the preferred catalytic metal could be that, because of the loss of the carboxylate side chain at the active site, a metal ion with a lower pKa, such as Mn^2+^, is then required to act as a Lewis acid instead for coordination.

10.1128/mbio.01834-22.7 FIG S4 Metal preference analysis of DrRuvC and EcRuvC. (A) Analysis of the metal preference of wild type DrRuvC and H139D mutant. Substrate HJ31 was used in this assay. 200 nM DNA was mixed with 1 μM protein and incubated with different concentrations (0.31, 0.625, 1.25, 2.5, 5, 10, and 20 mM) of Mg^2+^ or Mn^2+^ at 37°C for 30 min. The products were resolved by 8% native TBE-PAGE. (B) Analysis of the metal preference of wild type EcRuvC and D138H mutant. Substrate HJ31 was used in this assay. 200 nM DNA was mixed with 1 μM protein and incubated with different concentrations (0.31, 0.625, 1.25, 2.5, 5, 10, and 20 mM) of Mg^2+^ or Mn^2+^ at 37°C for 30 min. The products were resolved by 8% native TBE-PAGE. (C) The digestion fractions of the wild type EcRuvC and the D138H mutant from (B) were calculated by Image J from three repeats and displayed as a line chart using GraphPad Prism 9. Download FIG S4, PDF file, 0.3 MB. Copyright © 2022 Sun et al. 2022 Sun et al. https://creativecommons.org/licenses/by/4.0/ This content is distributed under the terms of the Creative Commons Attribution 4.0 International license.

### Analysis of the digestion efficiency and binding affinity of DrRuvC toward different DNA structures.

To find out whether DrRuvC is a structure-specific endonuclease, the digestion efficiencies and binding affinities of other DNA structures, such as duplex, nicked duplex, bulge, splayed duplex, overhang, flap, replication fork, Y-junction, immobile HJ, and nicked HJ, were tested and compared. All of the structured DNA possessed a shared 5′ end 6-FAM labeled strand (called a cleavage strand below) which contained the preferred cleavage sequence of DrRuvC, “TTCG”. All of the related sequences and structures are presented in Table S1B and Fig. S6.

10.1128/mbio.01834-22.2 TABLE S1 Oligonucleotides used in this study. (A) Primers used for cloning and mutagenesis. (B) Oligonucleotides used for digestion and binding assays. Download Table S1, PDF file, 1.9 MB. Copyright © 2022 Sun et al. 2022 Sun et al. https://creativecommons.org/licenses/by/4.0/ This content is distributed under the terms of the Creative Commons Attribution 4.0 International license.

10.1128/mbio.01834-22.9 FIG S6 The EMSA assays of DrRuvC toward different DNA structures. 100 nM of different substrates were incubated with different concentrations of DrRuvC (labeled on the top of each gel). The products were resolved by 5% TB-native gel. Download FIG S6, PDF file, 0.4 MB. Copyright © 2022 Sun et al. 2022 Sun et al. https://creativecommons.org/licenses/by/4.0/ This content is distributed under the terms of the Creative Commons Attribution 4.0 International license.

**FIG 6:**
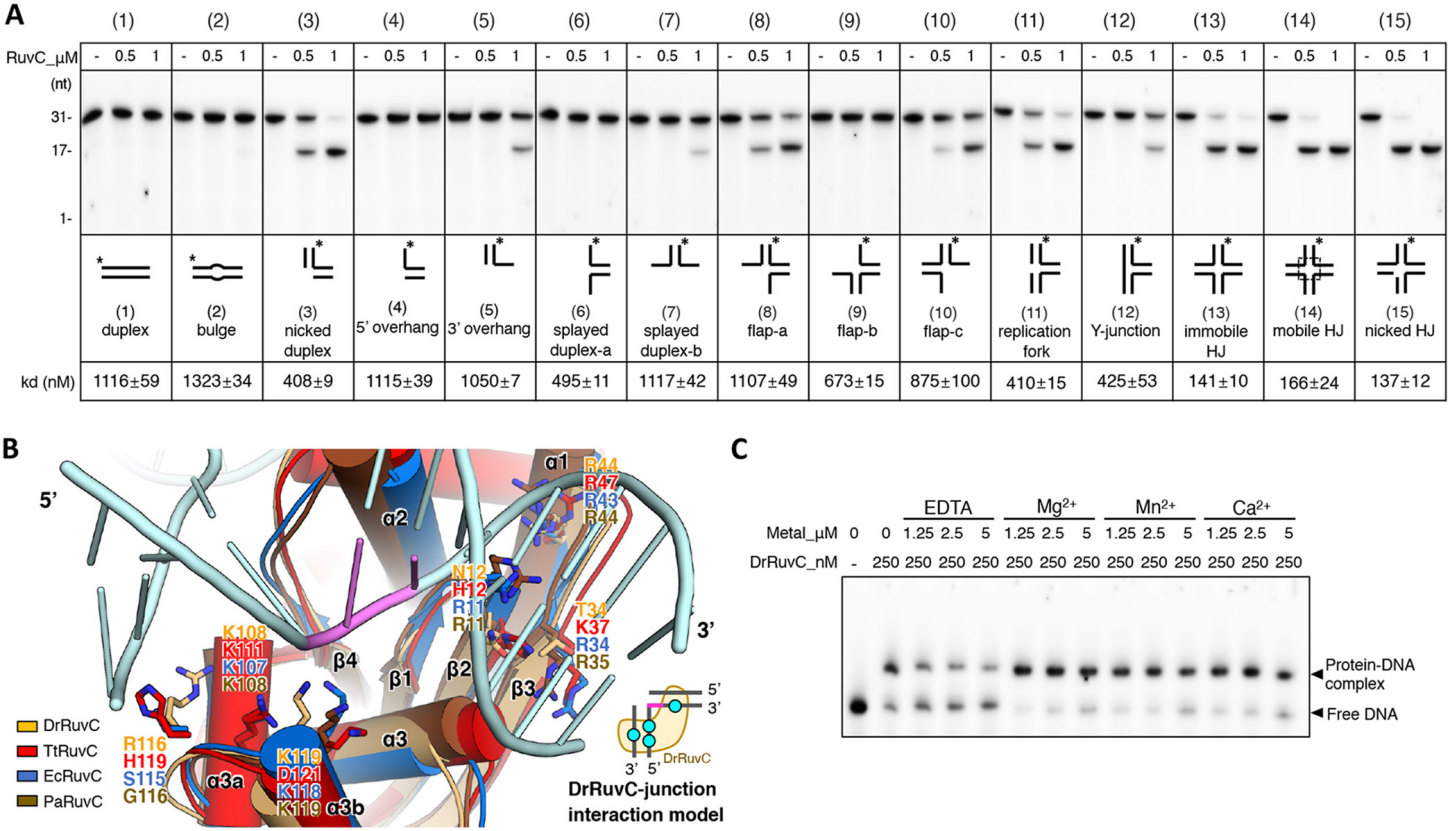
Analysis of the digestion efficiency and the binding affinity of DrRuvC. (A) The digestion efficiency and the binding affinity of DrRuvC toward different DNA structures. 200 nM substrate was mixed with 0.5 or 1 μM DrRuvC and 10 mM Mn^2+^ and then incubated at 37°C for 30 min. The products were resolved by 15% TBE-urea denaturing gels. Different DNA structures were shown below the gel. The corresponding binding K values of each structure were calculated from the EMSA results shown in Fig. S6. (B) The model of the DrRuvC-HJ complex. Based on the TtRuvC-HJ complex structure, the HJ substrate (cyan) was docked into the aligned binding motifs of DrRuvC (orange; this study), TtRuvC (red; PDB code: 6s16), EcRuvC (blue; PDB code: 1nmn), and PaRuvC (brown; PDB code: 6lw3) in PyMOL. The key putative residues for interactions are labeled and shown as sticks. A cartoon model for half of the DrRuvC-HJ complex was built in the right-hand corner. The cyan dots indicate the HJ binding sites on DrRuvC. The cleavage sites of HJ are colored pink. (C) The influence of divalent metal ions on the HJ binding affinity of DrRuvC. 100 nM HJ31x was incubated with 250 nM DrRuvC in the presence of different concentrations (0, 1.25, 2.5, and 5 mM) of EDTA, Mg^2+^, Mn^2+^, or Ca^2+^. The products were resolved by 5% TB-native gel.

Denaturing gel analysis indicated that the best substrates for DrRuvC are the mobile HJ and the nicked HJ (Fig. 6A). In the nicked HJ substrate, the symmetrical strand of the cleavage strand was pre-nicked and contained both hydroxyl groups on the 3′ and 5′ ends. It is worth noting that TtRuvC exhibited slightly stronger resolvase activity on a pre-nicked HJ substrate than on a non-pre-nicked HJ; however, phosphorylation of the 5′-end of the DNA at the nick was required (17). The immobile HJ substrate also exhibited a strong cut but was slightly weaker than that of the mobile HJ (Fig. 6A). As expected, no cleavage was seen on the DNA duplex. Endonuclease activity was detected on the replication fork and on the Y junction, as well (Fig. 6A). The sequence of the replication fork and the Y junction used in this assay are similar, except that the replication fork possesses a nick near the junction and makes the junction much more relaxed for DrRuvC binding (Fig. S6), which could be the reason why DrRuvC yielded more efficient cleavage on the replication fork than on the Y junction. Surprisingly, DrRuvC also displayed mild endonuclease activity on the 3′ overhang, some splayed duplex (splayed duplex-b), and some flap structures (flap-a and flap-c) (Fig. 6A). To sum up, what all these cleavable substrates have in common is that they contain a “TC” sequence on the cleavage strand near the junction, a relatively relaxed junction/core, and complementary strands that can form duplex arms together with the 5′ end of the cleavage strand (Fig. S6).

Next, we tested whether different cleavage efficiencies are due to different binding affinities (Fig. S6). As expected, mobile HJ, nicked HJ, and immobile HJ substrates can form obvious specific protein-DNA complex bands on the native gel (Fig. S6) with low K values (140 to 160 nM) (Fig. 6A). When increasing the protein concentrations, nonspecific protein-DNA complexes were formed and stacked in the gel hole. Specific protein-DNA complex bands were also detected on the splayed duplex, flap, replication fork, and Y-junction structures, although they were much weaker than those of the HJs (Fig. S6). Additionally, the binding K values of these substrates (400 to 1,100 nM) are much higher than those of HJ (Fig. 6A). In contrast, only nonspecific protein-DNA complex bands were detected on the duplex and bulge DNA substrates (Fig. S6), and their K values were greater than 1,100 nM (Fig. 6A). It is worth noting that although no obvious specific protein-DNA complex band was detected on the nicked duplex structure (Fig. S6), the K value of this substrate is relatively low (approximately 408 nM), similar to the K values of the replication fork and Y-junction structures (approximately 410 nM and 425 nM, respectively) (Fig. 6A).

According to the TtRuvC-HJ complex structure, there are two lysine residues, K111 and K122 (their corresponding residues in DrRuvC are K108 and K119, respectively), that assist in holding the 5′ end of the cleavage strand (Fig. 6B). It was reported that EcRuvC, when containing mutations of these corresponding residues (K107 and K118), completely lost its HJ resolution activity (37). There also exists one basic residue (R116 and H119 on DrRuvC and TtRuvC, respectively) on the loop between α3a and α3b, which might interact with the complementary strand of the 5′ end of the cleavage strand. However, there is no basic residue at a similar position on EcRuvC or on PaRuvC (Fig. 6B). However, the 3′ end of the cleavage strand is held by one conserved arginine residue on the α1 of the RuvCs (R44, R47, R43, and R44 on DrRuvC, TtRuvC, EcRuvC, and PaRuvC, respectively) (Fig. 6B). Other basic residues located at the N-terminal of β2 and the C-terminal of β3, such as H12 and K37 on TtRuvC, R11 and R34 on EcRuvC, and R11 and R35 on PaRuvC, might contribute to the 3′ end DNA binding, as well (Fig. 6B). However, DrRuvC does not have basic residues around a similar position, which implies that its interaction with the 3′ end of the cleavage DNA strand could be weaker than those of other RuvCs.

To sum up, our DNA digestion results indicate that the preferred substrate of DrRuvC is HJ. However, other DNA structures which contain the preferred cleavage sequence “TC” at the junction/loosen core and contain duplex arms beside the core, especially those containing the 5′ duplex arms, can also be digested by DrRuvC, albeit with much lower efficiencies. Those cleavable substrates suggest that the 3′ end duplex interaction is less critical for DrRuvC digestion, which is in agreement with the result from the protein-interaction analysis that the binding of the 3′ end duplex is weaker than the binding of the 5′ end duplex in DrRuvC (Fig. 6B).

Furthermore, the influence of metal ions on HJ binding affinity was also tested. To avoid the digestion, substrate HJ31x, which is similar to that of the HJ31 but displaces all of the “TC” with “TA” at the junction, was used. HJ31x exhibited a similar binding affinity with DrRuvC as did HJ31, but it cannot be digested by DrRuvC (Fig. S7). It was found that the appearance of low concentrations of divalent metal ions (1.25 or 2.5 mM) could help DrRuvC to bind to HJ, while EDTA exhibited inhibition effects (Fig. 6C).

10.1128/mbio.01834-22.10 FIG S7 The digestion and binding affinity comparisons of HJ31 and HJ31x. For the digestion assay, 100 nM HJ31 or HJ31x was incubated with different concentrations of DrRuvC (0, 62.5, 125, 250, 500, 1000, and 2000 nM) in the presence of 10 mM Mn^2+^ at 37°C for 30 min. The products were resolved by 15% denaturing gel. For the binding assay, 100 nM HJ31 or HJ31x was incubated with different concentrations of DrRuvC (0, 62.5, 125, 250, 500, 1000, and 2000 nM). The products were analyzed by 5% TB-native gel. Download FIG S7, PDF file, 0.4 MB. Copyright © 2022 Sun et al. 2022 Sun et al. https://creativecommons.org/licenses/by/4.0/ This content is distributed under the terms of the Creative Commons Attribution 4.0 International license.

### RNase activities of DrRuvC and DrYqgF.

**FIG 7:**
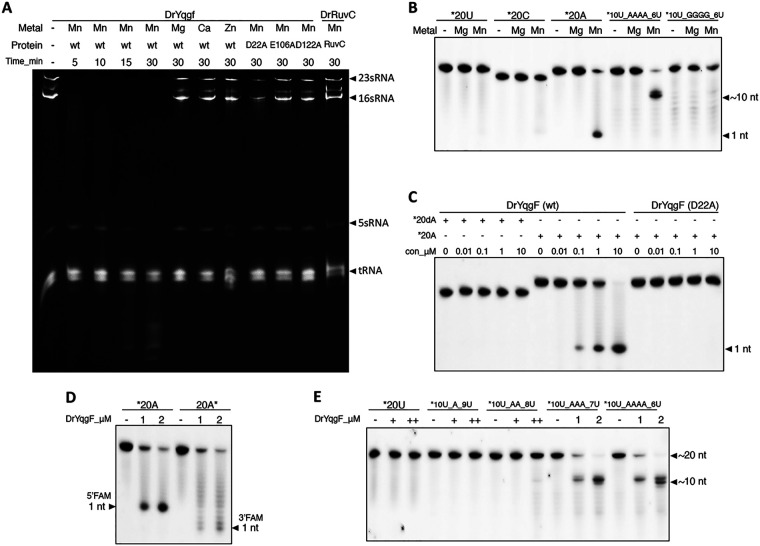
RNase activity analysis of DrYqgF. (A) The total RNA digestion assays of DrYqgF and DrRuvC. 2 μg of total RNA extracted from D. radiodurans were incubated with 1 μM wild type DrYqgF, site-directed DrYqgF mutants, and wild type DrRuvC in the presence of different kinds of metal ions. The products were resolved by 5% TBE-urea denaturing gel. (B) The analysis of the substrate sequence specificity of DrYqgF. 200 nM 20 nt RNA substrates with different sequences were incubated with 1 μM wild type DrYqgF and 10 mM metal (Mg^2+^ or Mn^2+^) at 37°C for 30 min. (C) Comparisons of the digestion efficiencies of wild type DrYqgF and D22A mutant on poly(dA) or poly(A) substrates. Different substrates (200 nM) were incubated with gradient concentrations (0, 0.01, 0.1, 1, and 10 μM) of protein and 10 mM Mn^2+^ at 37°C for 30 min. (D) Analysis of the exonuclease digestion direction of DrYqgF. 200 nM 20 nt poly(A) substrates, labeled at either the 5′ end or the 3′ end, were incubated with gradient concentrations (0, 1, and 2 μM) of DrYqgF and 10 mM Mn^2+^ at 37°C for 30 min. (E) Analysis of the preferred sequence for the endonuclease activity of DrYqgF. 200 nM 20 nt poly(U), which contained 0, 1, 2, 3, or 4 adenine bases within its sequence, were incubated with gradient concentrations (0, 1, and 2 μM) of DrYqgF and 10 mM Mn^2+^ at 37°C for 30 min. The products of (B), (C), (D) and (E) were resolved by 15% TBE-urea denaturing gel.

To identify whether DrYqgF has similar biochemical activities to EcYqgF, we first conducted RNase activity assays using purified total RNA from D. radiodurans as the substrates. Although EcYqgF only processed pre16S rRNA present in the 70S ribosome, DrYqgF exhibited robust RNase activity on purified total RNA in the presence of Mn^2+^ and preferred digesting 23S and 16S rRNAs (Fig. 7A). To confirm whether the RNase activity is mediated by the intrinsic activity of DrYqgF rather than by any contaminated RNase during purification, we mutated the putative key catalytic residues of DrYqgF into alanine and purified the mutants in the same way as wild type DrYqgF (Fig. S5). All of the DrYqgF mutants exhibited impaired (D22A) or blocked (E106A and D122A) RNase activity (Fig. 7A), suggesting that the RNase activity was mediated by the DrYqgF. Concurrently, DrRuvC showed no RNase activity (Fig. 7A).

10.1128/mbio.01834-22.8 FIG S5 Protein purity verifications. (A) The purities of purified DrRuvC, EcRuvC, and the related mutants were analyzed by 15% SDS-PAGE. (B) The purities of purified DrYqgF and the related mutants were analyzed by 15% SDS-PAGE. Download FIG S5, PDF file, 0.2 MB. Copyright © 2022 Sun et al. 2022 Sun et al. https://creativecommons.org/licenses/by/4.0/ This content is distributed under the terms of the Creative Commons Attribution 4.0 International license.

Furthermore, 20 nt ssRNA oligonucleotides with multiple sequences were used for an RNase activity analysis of DrYqgF. It was found that DrYqgF prefers to digest the poly(A) area within the RNA substrate in the presence of Mn^2+^ (Fig. 7B) in an exo/endonuclease manner. In contrast, the putative catalytic residue inactive mutant (D22A) did not show any activity over the poly(A) substrate (Fig. 7C). Meanwhile, DrYqgF did not digest 20 nt poly(dA), even at higher protein concentrations (Fig. 7C), which indicates that YqgF prefers RNA substrates. Moreover, no RNase activity was detected on short ssRNA (18 nt), dsRNA (18 bp), or an RNA/DNA hybrid (18 bp), whose sequences did not contain poly(A) (Fig. S8). Both the exonuclease and endonuclease activities of DrYqgF toward ssRNA were analyzed further. The direction of exonuclease was identified to be 5′–3′, since only 1 nt product was detected when the ssRNA was labeled at the 5′ end and since a series of band ladders appeared on the gel when the ssRNA was labeled at the 3′ end (Fig. 7D). As for the endonuclease activity, it seems that at least three consecutive adenine bases are required in the sequence for the efficient endonuclease activity of DrYqgF (Fig. 7E). Unlike MtYqgF, we did not detect any nuclease activity of DrYqgF over a variety of structured DNA substrates, even in the presence of ATP (data not shown). Furthermore, the structural analysis indicated that DrYqgF possesses no putative ATP binding or hydrolysis sites. DrYqgF did not exhibit any ATPase activity in the presence or absence of DNA/RNA oligonucleotides (data not shown).

10.1128/mbio.01834-22.11 FIG S8 The RNase activity analysis of drYqgF on short RNA substrates containing random sequence. 200 nM ssRNA, dsRNA, and dsRNA/DNA substrates with random sequences were incubated with 1 μM wild type DrYqgF and 10 mM metal (Mg^2+^ or Mn^2+^) at 37°C for 30 min. Reactions were carried out in the absence or presence of 5 mM ATP. The products were resolved by 15% TBE-urea denaturing gel. Download FIG S8, PDF file, 1.8 MB. Copyright © 2022 Sun et al. 2022 Sun et al. https://creativecommons.org/licenses/by/4.0/ This content is distributed under the terms of the Creative Commons Attribution 4.0 International license.

To sum up, our preliminary tests indicated that DrYqgF is a sequence-specific RNA 5′–3′ exo/endonuclease. However, it is still not known whether or not DrYqgF prefers structured RNA substrates.

## DISCUSSION

D. radiodurans possesses robust DNA damage repair efficiency, especially that of homologous recombination. Such efficiency is mediated either by modification of the inherent proteins or by the introduction of novel enzymes into the system. RuvC resolvases are conspicuously absent in the low-GC Gram-positive bacterial lineage, while the less characterized protein, YqgF, is represented in approximately 90% of bacterial lineages (8). Despite the low overall sequence similarity, the generally conserved topology and the same distributions of the two conserved acidic residues (located at β1 and α3, respectively) required for catalysis suggest that RuvC and YqgF diverged from a common ancestor and might possess similar enzymatic activity. Therefore, the YqgF family proteins could be alternative HJRs whose functions partially overlap those of RuvC. Low-GC Gram-positive bacteria which do not contain RuvC homologs usually encode another HJR, RecU (42). D. radiodurans encodes two putative HJRs, DrRuvC and DrYqgF but encodes no RecU homolog. Mutation assays showed that both DrRuvC and DrYqgF are essential proteins in D. radiodurans, which implies that their biological functions may not overlap. Our biochemical data suggests that only DrRuvC is a real HJR, while DrYqgF is an RNase.

The preferred cleavage site of DrRuvC was identified as a 5′-(G/C)TC↓(G/C)-3′ consensus sequence, different from that of EcRuvC and PaRuvC, which have been identified as 5′-(A/T)TT↓(G/C)-3′ and 5′-TTC-3′, respectively (18, 43). Since D. radiodurans has a genome with a high GC content (>65%) and displays robust homologous recombination efficiency (34, 35, 44), we consider that such a GC-rich cleavage site of DrRuvC evolved as an adaptation. It was reported that Mn^2+^ could relax the sequence specificity of EcRuvC (43). However, we did not notice that for DrRuvC, at least for the substrates (HJ31, HJ60, and HJ98) that we used in this study. Moreover, DrRuvC showed a preference for Mn^2+^ for its catalytic activity, which seems to be attributable to the evolutionary replacement of aspartic acid with histidine on the N terminus of α3. D. radiodurans accumulates high intracellular Mn(II), which facilitates its recovery from DNA damage (45), and a significant amount of a histidine-ligated Mn(II) metallopeptide/protein was detected in D. radiodurans (46). Therefore, we suggest that such evolutionary mutations of DrRuvC facilitate its regulation by the accumulated Mn(II) *in vivo*, making it a more efficient HJR in D. radiodurans.

During the revision of the manuscript, Qin et al. published the apo structural data and some biochemical features of DrRuvC (47). Although crystallized under different conditions, our DrRuvC structure can be superimposed well with theirs, which indicates a unanimous conformation of DrRuvC. They declared that Mn^2+^ is the preferred catalysis metal and that the presence of Mn^2+^ would increase the binding affinity of DrRuvC to HJ, which is in agreement with our results. Nevertheless, there are many contradictions. Qin et al. alleged that Mg^2+^ has a negative effect on the HJ binding affinity, while the effect of EDTA is modest (47). However, we noticed that EDTA could inhibit the binding of DrRuvC to HJ, while the addition of divalent metal ions (such as Mg^2+^, Mn^2+^, or Ca^2+^) enhances the binding affinity. It was reported that divalent cations or metal ions would induce the four-way DNA junction to undergo a conformational transition from an extended open square to a stacked-X structure (48–50). The bound HJ in the published TtRuvC-HJ complex structure is in a stacked-X shape (17, 20). Therefore, the existence of divalent metal ions might stabilize the stacked-X shape HJ, and that would help the RuvC to bind with HJ. Furthermore, inconsistent with their result that no nuclease activity was detected when Mg^2+^ was used (47), we did observe weak resolvase activity when high concentrations of Mg^2+^ appeared in the reaction system, which implied that DrRuvC is not a strict Mn^2+^-dependent nuclease. Furthermore, they showed that DrRuvC could not digest immobile HJ and Y junctions, and they concluded that the homologous core is strictly required for digestion (47). However, our data demonstrated that this is not the case. Although the digestion efficiencies and binding affinities are lower than those of mobile HJs, when the consensus sequence “TC” appeared at the junction area of the cleavage strand, endonuclease activities and specific protein-DNA interaction bands of DrRuvC toward a series of substrates, such as immobile HJ, replication fork, Y junction, nicked duplex, and flap, were also detected. We believe the reason that Qin et al. failed to detect the nuclease activity is that they did not used the preferred sequence of DrRuvC. In fact, in our assay, when replacing “TC” (preferred sequence) with “TA” (unpreferred sequence), even the mobile HJ (which contains a 6 nt homologous core) cannot be digested by DrRuvC.

Our biochemical data confirmed that DrYqgF has no nuclease activity on the HJ substrate, but it could digest 23S and 16S rRNAs from total extracted RNA and has a preference for the poly(A) sequence. However, the biological function of DrYqgF needs to be studied further. The *yqgF* gene could be removed in H. pylori and A. baylyi ADP1 (26, 27) but not in D. radiodurans, which is consistent with the indispensability of YqgF, as in E. coli (23), M. tuberculosis (24), and *S. typhimurium* (25). EcYqgF shares the same cleavage sites with EcRNase E and EcRNase G, which are mainly involved in 16S rRNA processing (30) and assist in the incorporation of ribosomal protein S1 into a ribosome via the processing of the 5′-end of pre16S rRNA (51). D. radiodurans naturally lacks RNase E and RNase G; therefore, whether DrYqgF plays an alternative function of RNase E or RNase G, as well as its participation in ribosome assembly, need to be confirmed. Although MtYqgF and BsYqgF were crystalized as dimers and DrYqgF was crystalized as a monomer, the cross-linking assay indicated that DrYqgF has a latent capacity to form a dimer. Dimerization assisted RuvC in digesting the HJ substrate symmetrically. We cannot rule out that dimerized DrYqgF could digest a specifically structured substrate symmetrically, as well. Similar to the RuvCs, DrYqgF possesses sequence preference on substrates. More structured RNA substrates need to be tested with DrYqgF to identify its exact role in the D. radiodurans.

## MATERIALS AND METHODS

### Sequence alignments.

Multiple sequence alignments were performed using Clustal Omega (52) and displayed by the online ENDscript server (53). Secondary structural elements are depicted according to the related PDB files.

### Mutant strain constructions.

Knockout of *drruvC* or *dryqgf* was carried out using a previously described deletion replacement method (40). The upstream and downstream target genes, which contain BamHI and HindIII digestion sites, were amplified by PCR. After BamHI and HindIII enzyme digestion, these segments were ligated to a kanamycin resistance cassette (*kan^r^*) (with BamHI and HindIII enzyme digestion, as well) and transformed into the wild type D. radiodurans strain R1. The mutant strains were screened with kanamycin-containing TGY plates (containing 1% tryptone, 0.5% yeast extract, 1% sodium chloride, 1.5% agar, and 20 mg/L kanamycin) and confirmed by PCR product analysis and sequencing.

### Cloning and site-directed mutagenesis.

The full-length gene encoding DrRuvC (residues 1 to 179aa) and drYqgF (residues 1 to 136aa) were amplified from D. radiodurans genomic DNA by PCR and cloned into a modified pET28a expression vector, pET28-HMT, which contains a fused N-terminal 6× His tag, an MBP-tag, and a TEV protease recognition site (His-MBP-TEV). The full-length gene encoding EcRuvC (residues 1 to 173aa) was amplified from E. coli K12 genomic DNA by PCR and cloned into the pET28a expression vector. Site-directed mutagenesis was performed with a QuikChange site-directed mutagenesis kit from Stratagene (La Jolla, CA), as described previously (54). The fidelities of the mutants were confirmed by sequencing. All of the successfully constructed vectors were transformed into E. coli Rossetta (DE3) strain (TransGen Biotech, Beijing). Primers were purchased from Sangon (Shanghai, China). A list of the primers used for cloning and mutagenesis is provided in Table S1A.

### Protein expression and purification.

The DrRuvC, DrYqgF, and other mutant variants were expressed and purified in the same way. In brief, transformed E. coli Rossetta (DE3) clones were grown at 37°C in LB medium containing 50 mg/mL kanamycin to an optical density at 600 nm of 0.6 to 0.8. Protein expression was induced at 30°C for 5 h by adding isopropyl-β-d-thioga-lactopyranoside (IPTG) with a final concentration of 0.8 mM. After harvesting, cells were resuspended in lysis buffer (20 mM Tris [pH 8.0], 1 M NaCl, 0.5 mM Tris [2-carboxyethyl] phosphine [TCEP], and 5 mM imidazole), lysed by sonication, and centrifuged at 20,000 × *g* and 4°C for 60 min. The supernatant was purified with a HisTrap HP column (GE Healthcare, Fairfield, CT), equilibrated with lysis buffer, washed with 30 mM imidazole, and eluted with 200 mM imidazole. After TEV-tag-removal using the TEV protease, the protein was dialyzed into buffer B (20 mM Tris [pH 8.0], 100 mM NaCl, and 0.5 mM TCEP) and reloaded onto the HisTrap HP column (GE Healthcare) to remove the uncleaved protein and TEV protease. The flowthrough fractions were collected and loaded onto a Heparin HP column (GE Healthcare) that was preequilibrated with buffer B. Fractions containing DrRuvC or DrYqgF protein were eluted with a linear gradient from 100 mM to 600 mM NaCl. The EcRuvC and related mutant were expressed and sonicated in the same way as was DrRuvC. The supernatant was purified with a HisTrap HP column (GE Healthcare, Fairfield, CT), equilibrated with lysis buffer, washed with 30 mM imidazole, and eluted with 300 mM imidazole. The collected protein was dialyzed into buffer B (20 mM Tris [pH 8.0], 100 mM NaCl, and 0.5 mM TCEP) and loaded onto a Heparin HP column (GE Healthcare) preequilibrated with buffer B. Fractions containing EcRuvC were eluted with a linear gradient from 100 mM to 600 mM NaCl. All of the proteins were finally purified with a Superdex 75 10/300 GL column (GE Healthcare) with buffer C (20 mM Tris [pH 8.0], 100 mM NaCl, and 0.5 mM TCEP) and stored at −80°C.

### Gel filtration chromatography.

Analytical size exclusion chromatography was performed using a Superdex 75 10/300GL column (GE Healthcare) with a flow rate of 0.3 mL/min. The column was equilibrated with a buffer containing 20 mM Tris (pH 8.0), 200 mM NaCl, and 0.5 mM TCEP. 100 μL of purified protein (2 mg/mL) were loaded. The fractions were monitored by UV absorbance at 280 nm. A set of protein standards of known molecular mass, such as aprotinin (6.5 kDa), RNase A (13.7 kDa), carbonic anhydrase (29 kDa), ovalbumin (43 kDa), and conalbumin (75 kDa), were used to construct the standard curve. The sizes of the calibration proteins at the positions where they eluted were marked on the *x* axis, based on the method described for the gel filtration calibration kit HMW (GE Healthcare). The elution volume (V_e_) corresponding to the protein peak was determined, and the molecular weight was calculated via interpolation on the standard curve.

### Chemical cross-linking.

Purified DrRuvC solution (0.1 mM) or DrYqgF (0.1 mM) was dialyzed against a buffer containing 20 mM HEPES (pH 8.0), 100 mM NaCl, and 10% glycerol, and it was treated with the indicated amounts of freshly diluted glutaraldehyde (0.01, 0.02, 0.04, 0.08, 0.16, and 0.32%) (Sangon, Shanghai) or bis-sulfo-succinimidyl suberate (BS3) (0.125, 0.25, 0. 5, 1, 2, and 4 mM) (Thermo Fisher Scientific, USA). After incubation at 25°C for 30 min, samples were treated with amine-containing quenching buffer (Tris [pH 8.0], with a final concentration to 100 mM) and then further incubated at 25°C for 10 min. Then, SDS-PAGE sample loading buffer was added. Samples were boiled for 10 min and analyzed by 12% (for DrRuvC) or 15% (for DrYqgF) SDS-polyacrylamide gel electrophoresis. The cross-linked and non-cross-linked bands were visualized via staining with Coomassie Brilliant Blue.

### Crystallization and structure determination.

Crystallization trials were performed by the sitting drop vapor diffusion method at 289 K. Fresh purified protein was concentrated (DrRuvC was concentrated to ~5 mg/mL, and DrYqgF was concentrated to ~10 mg/mL) and centrifuged at 15,000 × *g* for 5 min at 4°C to remove insoluble fractions before crystallization. After a series of screening tests and optimizations, the best crystals of DrRuvC were obtained under the conditions of 0.2 M MgCl_2_, 0.1 M HEPES (pH 7.8), and 20% PEG 3350. The best crystals of DrYqgF were obtained under the conditions of 0.1 M MgCl_2_, 0.1 M HEPES (pH 7.0), and 15% PEG4000. The crystals were flash frozen in liquid nitrogen. X-ray diffraction data were collected on beamline BL02U1 at the Shanghai Synchrotron Radiation Facility (Shanghai, China) and integrated and scaled with the XDS suite (55). The DrRuvC structure and the DrYqgF structure were determined by molecular replacement using the TtRuvC structure (PDB code: 4EP4) and the EcYqgF structure (PDB code: 1OVQ) as the search models in CCP4, respectively, followed by rigid body refinement using REFMAC5 (56). Structures were refined using PHENIX (57) and were interspersed with manual model building using COOT (58). All residues were in the most favorable and allowed regions of the Ramachandran plot. All structural figures were created by PyMOL. The statistics for the data collection and refinement are listed in Table 1.

### DNA and RNA substrates.

All of the oligonucleotide DNA and RNA (the sequences are listed in Table S1B) were purchased from Sangon (Shanghai, China), with or without the 5′-ends labeled with 6-carboxfluorescein (6-FAM). Oligos FAM-J31-1, J31-2, J31-3, and J31-4 can be annealed into substrate HJ31. Oligos FAM-J98-1, J98-2, J98-3, and J98-4 can be annealed into substrate HJ98a. Oligos J98-1, FAM-J98-2, J98-3, and J98-4 can be annealed into substrate HJ98b. Oligos FAM-J60-1, J60-2, J60-3, and J60-4 can be annealed into substrate HJ60a. Oligos J60-1, FAM-J60-2, J60-3, and J60-4 can be annealed into substrate HJ60b. Oligos J24-N^1^N^2^N^3^N^4^-1, J24-D^1^D^2^D^3^D^4^-2, J24-N^1^N^2^N^3^N^4^-3, and J24-D^1^D^2^D^3^D^4^-4 can be annealed into substrate HJ24-N^1^N^2^N^3^N^4^. Oligos FAM-ssRNA and ssRNA-R can be annealed into dsRNA substrate. Oligos FAM-ssRNA and ssDNA-R can be annealed into dsRNA/DNA substrate. The annealing reactions were carried out by heating the oligonucleotide mixture at 95°C for 5 min and then following with slow cooling to room temperature in annealing buffer (20 mM Tris [pH 8.0], 50 mM NaCl, and 0.5 mM TCEP). As for annealing of the FAM-labeled substrates, labeled and unlabeled oligonucleotides were mixed in a 1:1.5 molar ratio. As for the annealing of the unlabeled substrates, equal molar oligonucleotides were used. The annealed substrates were further checked using native polyacrylamide gel to analyze the purity.

The total RNA of D. radiodurans was isolated using TRIzol Reagent according to the manufacturer’s protocol (Ambion, USA). Briefly, D. radiodurans was grown in TGY medium until the optical density at 600 nm reached ~0.6. Then, cells were centrifuged and resuspended in 1 mL of TRIzol solution. Phenol-chloroform was then added, and the tubes were shaken for 15 s. The mixed samples were then incubated for 15 min at room temperature before being centrifuged at 12,000 × *g* at 4°C for 15 min to derive the three distinct phases. The upper colorless phase was transferred into a new tube, and 150 μL of isopropanol with 1 μL of glycogen were added. The sample was mixed briefly and incubated at room temperature for 10 min. The sample was then centrifuged at 12,000 × *g* at 4°C for 10 min, and the supernatant was discarded. The remaining white RNA pellet was washed with 300 μL of 75% ethanol and then spun down at 7,500 × *g* for 5 min. The ethanol was then discarded, and the pellet was air-dried for 5 to 10 min until it turned transparent and then redissolved in 20 μL of RNase-free water. Nanodrop (Thermo Fisher Scientific, USA) was used to measure the RNA concentrations.

### Endonuclease activity assays.

For the HJ resolvase assays, typical reaction mixtures (20 μL) contain 200 nM DNA, 20 mM Tris (pH 8.0), 10 μg/mL bovine serum albumin, 1 mM DTT, 10 mM MgCl_2_ (or 10 mM MnCl_2_, or 10 mM CaCl_2_, or 10 mM ZnSO_4_), 5% glycerol, and proteins (DrRuvC, DrYqgF, or EcRuvC) at the indicated concentrations. Reaction mixtures were incubated at 37°C for 30 min and terminated by the addition of an equal volume of stop solution (containing 20 mM EDTA, 10 mg/mL proteinase K, and an additional 98% formamide for the denaturing gel), followed by further incubation at 37°C for 20 min and boiling for 20 min for the denaturing gel, exclusively. Products were separated on 10% Tris-borate-EDTA (TBE)-native PAGE or 12% (or 15%) TBE-urea denaturing PAGE (containing 7 M urea). The gels containing 6-FAM labeled substrates were imaged using the fluorescence mode (FAM) on a ChemiScope6100 (Clinx Science Instruments, Shanghai). As for the unlabeled substrates, 2.5 μM DNA were mixed with 5 μM DrRuvC, and the gels were finally stained by Stains-all (Sangon, China). Bands were analyzed using ImageJ software (National Institutes of Health, USA) and GraphPad Prism 9 (San Diego, USA) (40), if necessary.

For the total RNA digestion assays, typical reaction mixtures (20 μL) contained 2 μg of total RNA from D. radiodurans, 20 mM Tris (pH 8.0), 10 μg/mL BSA, 1 mM DTT, 10 mM MgCl_2_ (or 10 mM MnCl_2_, or 10 mM CaCl_2_, or 10 mM ZnSO_4_), 5% glycerol, 1 U RNase inhibitor (Sangon, Shanghai), and 1 μM DrRuvC (or DrYqgF). The reaction mixtures were incubated at 37°C for 5 to 30 min and were terminated by the addition of an equal volume of stop solution (20 mM EDTA, 10 mg/mL proteinase K, and 98% formamide), followed by boiling for 20 min. Products were separated in 5% TBE-urea denaturing PAGE (containing 7 M urea), stained with Grblue (Generay Biotech, Shanghai), and then imaged using the UV mode on a ChemiScope6100 (Clinx Science Instruments, Shanghai).

For the short RNA digestion assays, typical reaction mixtures (10 μL) contained 200 nM substrate, 10 μg/mL BSA, 1 mM DTT, 10 mM MgCl_2_ (or 10 mM MnCl_2_), 5% glycerol, and 0 to 10 μM wild type DrYqgF (or D22A mutant). Reaction mixtures were incubated at 37°C for 30 min and were terminated by the addition of an equal volume of stop solution (containing 20 mM EDTA, 10 mg/mL proteinase K, and 98% formamide), followed by boiling for 20 min. Products were separated on 15% TBE-urea denaturing PAGE (containing 7 M urea). The gels were imaged using the fluorescence mode (FAM) on a ChemiScope6100 (Clinx Science Instruments, Shanghai).

### DNA binding assays.

DNA binding affinities were analyzed according to a previously reported method (59), with some modifications. 100 nM 6-FAM labeled substrates were mixed with different concentrations of DrRuvC in a 10 μL reaction volume containing 50 mM TRIS-HCl (pH 8.0), 125 mM NaCl, 10 μg/mL BSA, 1 mM TCEP, and 5% (vol/vol) glycerol. Different concentrations of metal ions or EDTA were added to the mixture, if necessary. After incubation at 30°C for 10 min, the samples were separated on 5% native polyacrylamide gels in 0.5× Tris-borate buffer. Gels were imaged using the fluorescence mode (FAM) on a ChemiScope6100 (Clinx Science Instruments, Shanghai). The binding fractions were calculated using Image J from three repeats, and the K values were calculated using GraphPad Prism 9.

### Data availability.

Atomic coordinates and structure factors for the reported crystal structures have been deposited with the Protein Data Bank under accession numbers 7W8D and 7W89.
