# Supplementary material for: Biochemical and Structural Study of RuvC and YqgF from Deinococcus radiodurans
Source: mBio. 2022 Aug 24;13(5):e01834-22. doi: 10.1128/mbio.01834-22 (PMC9601230; doi:10.1128/mbio.01834-22)
Supplement: TABLE S2 [file mbio.01834-22-s0002.pdf]

Supplementary table S2. The percentages of amino acids sequence identities.

(A) The percentage of amino acids sequence identity between the RuvC resolvases.

	drRuvC	ttRuvC	ecRuvC	paRuvC	abRuvC	mtRuvC	hpRuvC
drRuvC	100.00	44.44	32.94	32.75	31.82	35.20	32.26
ttRuvC	44.44	100.00	34.78	37.27	34.16	33.54	29.30
ecRuvC	32.94	34.78	100.00	55.49	56.07	34.88	32.69
paRuvC	32.75	37.27	55.49	100.00	54.02	32.95	33.33
abRuvC	31.82	34.16	56.07	54.02	100.00	32.78	36.54
mtRuvC	35.20	33.54	34.88	32.95	32.78	100.00	32.48
hpRuvC	32.26	29.30	32.69	33.33	36.54	32.48	100.00

The percentage of sequence identities were calculated by Clustal Omega webserver. Names of species are dr, *Deinococcus radiodurans*; tt, *Thermus thermophilus* HB8; ec, *Escherichia coli*; pa, *Pseudomonas aeruginosa*; ab, *Acinetobacter baylyi*mt; *Mycobacterium tuberculosis*; hp, *Helicobacter pylori*.

(B) The percentage of amino acids sequence identity between the YqgFs.

	drYqgf	ttYqgf	ecYqgf	paYqgf	abYqgf	mtYqgf	hpYqgf	bsYqgf
drYqgf	100.00	45.45	29.27	30.47	25.00	29.41	22.61	26.45
ttYqgf	45.45	100.00	28.89	27.41	25.19	30.37	2812	26.67
ecYqgf	29.27	28.89	100.00	48.55	39.86	28.26	29.69	32.35
paYqgf	30.47	27.41	48.55	100.00	38.19	27.78	20.16	33.09
abYqgf	25.00	25.19	39.86	38.19	100.00	20.41	20.61	22.06
mtYqgf	29.41	30.37	28.26	27.78	20.41	100.00	21.37	33.82
hpYqgf	22.61	28.12	29.69	20.16	20.61	21.37	100.00	21.09
bsYqgf	26.45	26.67	32.35	33.09	22.06	33.82	21.09	100.00

The percentage of sequence identities were calculated by Clustal Omega webserver. Names of species are dr, *Deinococcus radiodurans*; tt, *Thermus thermophilus* HB8; ec, *Escherichia coli*; pa, *Pseudomonas aeruginosa*; ab, *Acinetobacter baylyi*mt; *Mycobacterium tuberculosis*; hp, *Helicobacter pylori*; bs, *Bacillus subtilis*.
