# Supplementary material for: Biochemical and Structural Study of RuvC and YqgF from Deinococcus radiodurans
Source: mBio. 2022 Aug 24;13(5):e01834-22. doi: 10.1128/mbio.01834-22 (PMC9601230; doi:10.1128/mbio.01834-22)
Supplement: FIG S1 [file mbio.01834-22-s0003.pdf]

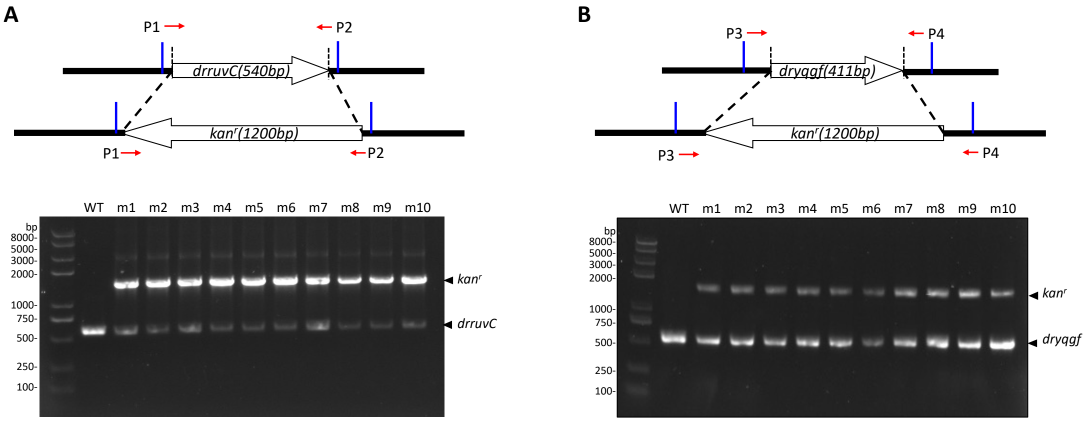

Supplementary figure S1. Construction and verification of the deletion mutants. Upper, schematic of the constructions of *drruvc* **(A)** or *dryqgf* **(B)** deletion mutants. The map of *drruvC* **(A)** or *dryqgf* **(B)** in the *D. radiodurans* chromosome before (top) or after (bottom) replacement with a *kanamycin* resistance cassette are shown. Lower, ethidium bromide-stained agarose gel illustrating that the mutants carry heterozygous deletions of *drruvC* **(A)** or *dryqgf* **(B)**. WT, wild type strain. m1-10, different mutant strains.
