# Supplementary material for: Biochemical and Structural Study of RuvC and YqgF from Deinococcus radiodurans
Source: mBio. 2022 Aug 24;13(5):e01834-22. doi: 10.1128/mbio.01834-22 (PMC9601230; doi:10.1128/mbio.01834-22)
Supplement: FIG S2 [file mbio.01834-22-s0004.pdf]

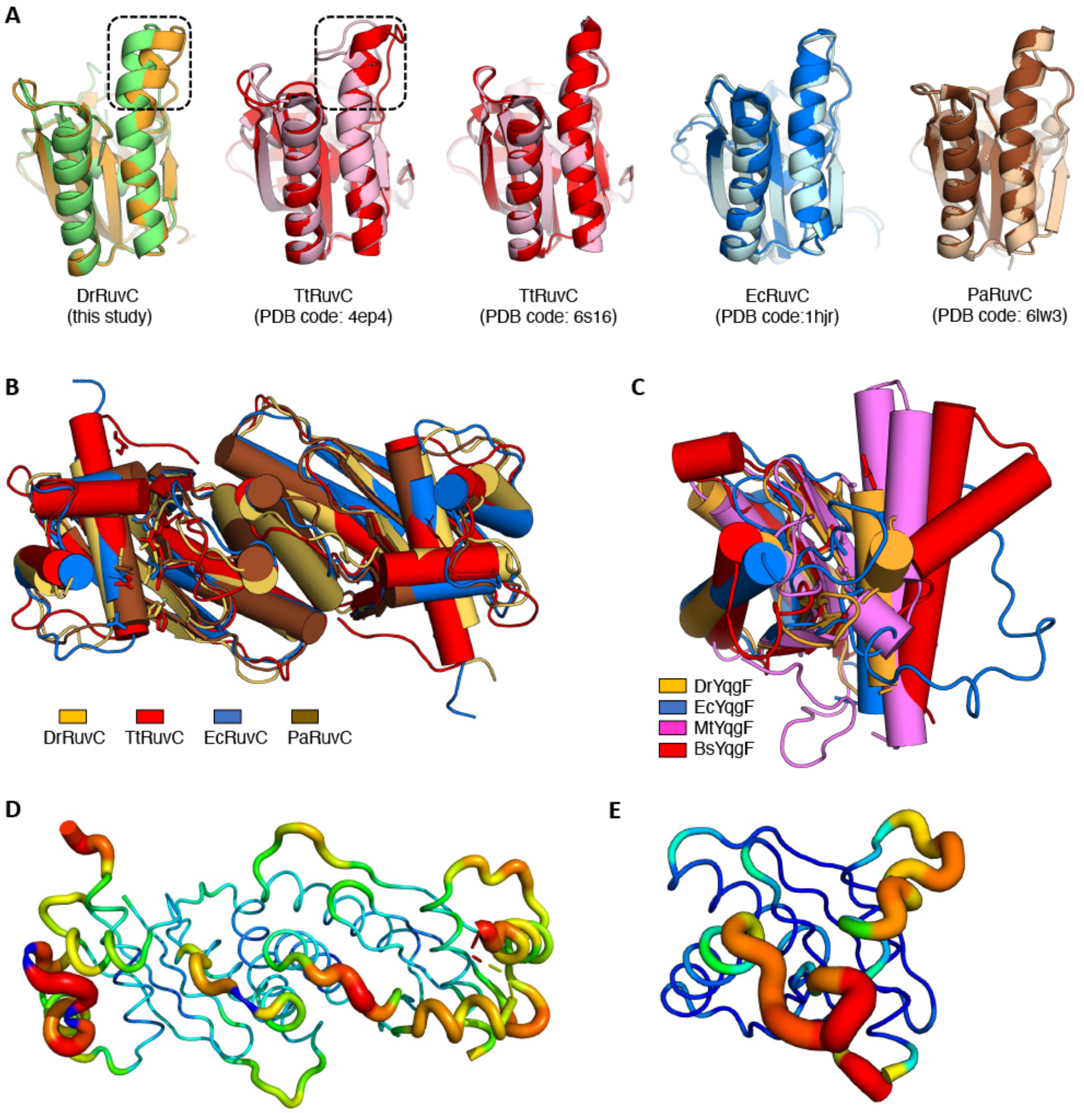

Supplementary figure S2. Structural analysis of RuvCs and YqgFs.
(A) Superimpositions of the two molecules from DrRuvC apo homodimer structure (this study), TtRuvC apo homodimer structure (PDB code: 4ep4), EcRuvC homodimer structure (PDB code:1hjr), PaRuvC homodimer structure (PDB code: 6lw3), and TtRuvC homodimer–HJ complex structure (PDB code: 6s16). Each molecule of the dimer was shown as different color. **(B)** Superimpositions of the overall structures of RuvC dimers. DrRuvC (orange; this study), TtRuvC (red; PDB code: 4ep4), EcRuvC (blue; PDB code:1nmn), and PaRuvC (brown; PDB code:6lw3) were shown as cartoon and superimposed. **(C)** Superimposition of the overall structures of YqgF. DrYqgF (orange; this study), EcYqgF (blue; PDB code: 1ovq), MtYqgF (violet; PDB code:7dr3), and BsYqgF (red; PDB code:1vhx) were shown as cartoon and superimposed. **(D)** The temperature factor of DrRuvC dimer. The temperature factor was calculated by PyMOL and the structure was shown as cartoon putty and colored according to the spectrum of the temperature factor values (the higher the factor value, the bigger size of the cartoon putty and the warmer of the color). The direction of view is the same as B. E, The temperature factor of DrYqgF. All the settings are the same as D and the direction of the view is the same as C.
