# Supplementary material for: Biochemical and Structural Study of RuvC and YqgF from Deinococcus radiodurans
Source: mBio. 2022 Aug 24;13(5):e01834-22. doi: 10.1128/mbio.01834-22 (PMC9601230; doi:10.1128/mbio.01834-22)
Supplement: FIG S4 [file mbio.01834-22-s0006.pdf]

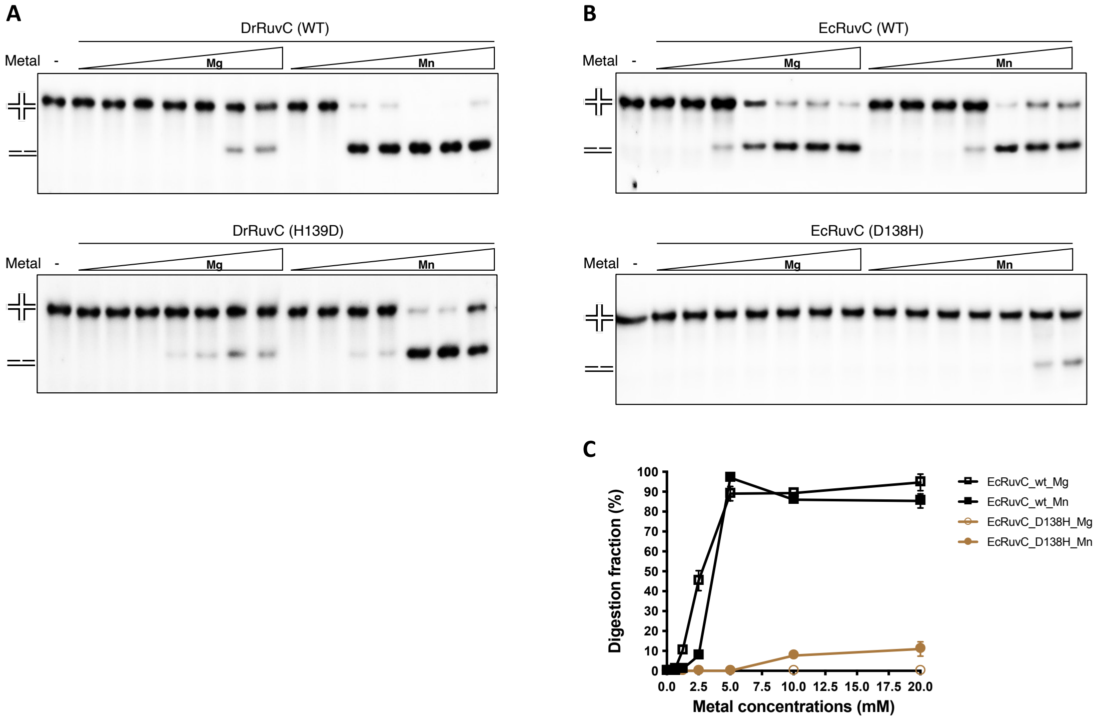

Supplementary figure S4. Metal preference analysis of DrRuvC and EcRuvC.

(A) Analysis of the metal preference of wild type DrRuvC and H139D mutant. Substrate HJ31 was used in this assay. 200 nM DNA was mixed with 1 μM protein and incubated with different concentrations (0.31, 0.625, 1.25, 2.5, 5, 10, and 20 mM) of Mg^{2+} or Mn^{2+} at 37°C for 30 min. The products were resolved by 8% native TBE-PAGE. **(B)** Analysis of the metal preference of wild type EcRuvC and D138H mutant. Substrate HJ31 was used in this assay. 200 nM DNA was mixed with 1 μM protein and incubated with different concentrations (0.31, 0.625, 1.25, 2.5, 5, 10, and 20 mM) of Mg^{2+} or Mn^{2+} at 37°C for 30 min. The products were resolved by 8% native TBE-PAGE. **(C)** The digestion fractions of wild type EcRuvC and D138H mutant from B were calculated by Image J from three repeats, and displayed as line chart using GraphPad Prism 9.
