# Supplementary material for: Biochemical and Structural Study of RuvC and YqgF from Deinococcus radiodurans
Source: mBio. 2022 Aug 24;13(5):e01834-22. doi: 10.1128/mbio.01834-22 (PMC9601230; doi:10.1128/mbio.01834-22)
Supplement: TABLE S1 [file mbio.01834-22-s0001.pdf]

Supplementary table S1. Oligos used in this study.

(A) Primers used for cloning and mutagenesis.

Primers	Sequence (5′→3′)
drRuvC_FN	ttttttcatatgagggttctggggat
drRuvC_RB	tttggatcctcagcgccgcagcgggg
drYqgf_FN	ttttttcatatgctcgcccggatgtc
drYqgf_RB	tttggatccttacctcatcagccaca
ecRuvC_FN	tacttccaaggtcatatggctattattctcggc
ecRuvC_RB	gagctcgaattcggatccttaacgcagtcgccct
ecRuvC_D138H	gcatcggcggcatgcgcctgtggatta
ecRuvC_D138H	taatccacaggcgcatgccgccgatgc
RuvC_D7A_F	cagaccgggggcaatccccagaaccc
RuvC_D7A_R	gggttctggggattgcccccggtctg
RuvC_E67A_F	cagaatctggtccgcgatcgccaccgc
RuvC_E67A_R	gcggtggcgatcgcggaccagattctg
RuvC_H139A_F	gcgtcggcggcggcgttgttgaacagctcgc
RuvC_H139A_R	gcgagctgttcaacaacgccgccgccgacgc
RuvC_H139D_F	gtcggcggcgtcgttgttgaacagctc
RuvC_H139D_R	gagctgttcaacaacgacgccgccgac
RuvC_D142A_F	gagccagcgcggcggcggcgtgg
RuvC_D142A_R	ccacgccgccgccgcgctggctc
Yqgf_D22A_F	cgacttgctcacggcgagggccagcac
Yqgf_D22A_R	gtgctggccctcgccgtgagcaagtcg
Yqgf_E106A_F	ctgcgtggtaaagcgtgcgtcctgatactcgac
Yqgf_E106A_R	gtcgagtatcaggacgcacgctttaccacgcag
Yqgf_D122A_F	gctgccgcctcagcctcatcggcag
Yqgf_D122A_R	ctgccgatgaggctgaggcggcagc

(B) Oligos used for digestion and binding assays.

Name	Sequence (5′→3′)
FAM-J31-1	FAM-GCTAGCCACAGCCATTCGTACGATTGCGGGA
J31-2	TCCCGCAATCGTACGAAACCGAGCACGATCT
J31-3	AGATCGTGCTCGGTTTCGTACCAGATGCCAT
J31-4	ATGGCATCTGGTACGAATGGCTGTGGCTAGC
J31-2m	TCCCGCAATCGTCCGTTTCCGAGCACGATCT
J31-3m	AGATCGTGCTCGGAAAGCCTGCAGATGCCAT
J31-4m	ATGGCATCTGCAGGCAATGGCTGTGGCTAGC
J31-5	TCCCGCAATCGTACG
J31-6	AGATCGTGCTCGGAAA
J31-7	AATGGCTGTGGCTAGC
J31-8	AGATCGTGCTCGGAAAAATGGCTGTGGCTAGC
J31-9	GCCTGCAGATGCCAT
J31-10	TCCCGCAATCGTACGAATGGCTGTGGCTAGC
J31-11	TCCCGCAATCGTATTTTTGGCTGTGGCTAGC
FAM-J31x-1	FAM-GCTAGCCACAGCCATTATGACTATTGCGGGA

J31x-2	TCCCGCAATAGTACTAAACCGAGCACGATCT
J31x-3	AGATCGTGCTCGGTTTAGTACCAGATGCCAT
J31x-4	ATGGCATCTGGTACTAATGGCTGTGGCTAGC
FAM-J98-1	FAM-TTCTAAGACCCTGAACCACTCGGGAAATAACAAGATTTCATCTATGACCAGTACGAGCTTAGGTTGTCCTGGCCCGCGTGCAAAGGATGACAGAAGCA
J98-1	TTCTAAGACCCTGAACCACTCGGGAAATAACAAGATTTCATCTATGACCAGTACGAGCTTAGGTTGTCCTGGCCCGCGTGCAAAGGATGACAGAAGCA
FAM-J98-2	FAM-TGCTTCTGTCATCCTTTGCACGCGGGCCAGGACAACCTAAGCTCGTACTGGTCATAGATGAAATCTTGTTATTTCCCGAGTGTCAATCCATACTTCGT
J98-2	TGCTTCTGTCATCCTTTGCACGCGGGCCAGGACAACCTAAGCTCGTACTGGTCATAGATGAAATCTTGTTATTTCCCGAGTGTCAATCCATACTTCGT
J98-3	ACGAAGTATGGATTGACACTCGGGAAATAACAAGATTTCATCTATGACCAGTACGAGCTTAGGTTGTCCTGGCCCGCGTGCAGATTCTCAAGATGACT
J98-4	AGTCATCTTGAGAATCTGCACGCGGGCCAGGACAACCTAAGCTCGTACTGGTCATAGATGAAATCTTGTTATTTCCCGAGTGGTTCAGGGTCTTAGAA
FAM-J60-1	FAM-TGCCGAATTCTACCAGTGCTTTGTATACCGTATACGGACATCTTGCCCACCTGCAGGTTC
J60-1	TGCCGAATTCTACCAGTGCTTTGTATACCGTATACGGACATCTTGCCCACCTGCAGGTTC
FAM-J60-2	FAM-GAACCTGCAGGTGGGCAAGATGTCCGTATACGGTATACAAAGTAATCGTCAAGCTTATGC
J60-2	GAACCTGCAGGTGGGCAAGATGTCCGTATACGGTATACAAAGTAATCGTCAAGCTTATGC
J60-3	GCATAAGCTTGACGATTACTTTGTATACCGTATACGGACATGCTGTCTAGAGGATCCGAC
J60-4	GTCGGATCCTCTAGACAGCATGTCCGTATACGGTATACAAAGCACTGGTAGAATTCGGCA
J24-TTCG-1	GCCACAGCCATTCGCCCATTGCGT
J24-TTCG-2	ACGCAATGGGCGAAACCGAGCACG
J24-TTCG-3	CGTGCTCGGTTTCGTGCAGATGCC
J24-TTCG-4	GGCATCTGCACGAATGGCTGTGGC
J24-ATCG-1	GCCACAGCCAATCGCCCATTGCGT
J24-ATCG-2	ACGCAATGGGCGATACCGAGCACG
J24-ATCG-3	CGTGCTCGGTATCGTGCAGATGCC
J24-ATCG-4	GGCATCTGCACGATTGGCTGTGGC
J24-CTCG-1	GCCACAGCCACTCGCCCATTGCGT
J24-CTCG-2	ACGCAATGGGCGAGACCGAGCACG
J24-CTCG-3	CGTGCTCGGTCTCGTGCAGATGCC
J24-CTCG-4	GGCATCTGCACGAGTGGCTGTGGC
J24-GTCG-1	GCCACAGCCAGTCGCCCATTGCGT
J24-GTCG-2	ACGCAATGGGCGACACCGAGCACG
J24-GTCG-3	CGTGCTCGGTGTCGTGCAGATGCC
J24-GTCG-4	GGCATCTGCACGACTGGCTGTGGC

J24-TTCC-1	GCCACAGCCATTCCCCCATTGCGT
J24-TTCC-2	ACGCAATGGGGGAAACCGAGCACG
J24-TTCC-3	CGTGCTCGGTTTCCTGCAGATGCC
J24-TTCC-4	GGCATCTGCAGGAATGGCTGTGGC
J24-ATCC-1	GCCACAGCCAATCCCCCATTGCGT
J24-ATCC-2	ACGCAATGGGGGATACCGAGCACG
J24-ATCC-3	CGTGCTCGGTATCCTGCAGATGCC
J24-ATCC-4	GGCATCTGCAGGATTGGCTGTGGC
J24-CTCC-1	GCCACAGCCACTCCCCCATTGCGT
J24-CTCC-2	ACGCAATGGGGGAGACCGAGCACG
J24-CTCC-3	CGTGCTCGGTCTCCTGCAGATGCC
J24-CTCC-4	GGCATCTGCAGGAGTGGCTGTGGC
J24-GTCC-1	GCCACAGCCAGTCCCCCATTGCGT
J24-GTCC-2	ACGCAATGGGGGACACCGAGCACG
J24-GTCC-3	CGTGCTCGGTGTCCTGCAGATGCC
J24-GTCC-4	GGCATCTGCAGGACTGGCTGTGGC
J24-TTCA-1	GCCACAGCCATTCACCCATTGCGT
J24-TTCA-2	ACGCAATGGGTGAAACCGAGCACG
J24-TTCA-3	CGTGCTCGGTTTCATGCAGATGCC
J24-TTCA-4	GGCATCTGCATGAATGGCTGTGGC
J24-ATCA-1	GCCACAGCCAATCACCCATTGCGT
J24-ATCA-2	ACGCAATGGGTGATACCGAGCACG
J24-ATCA-3	CGTGCTCGGTATCATGCAGATGCC
J24-ATCA-4	GGCATCTGCATGATTGGCTGTGGC
J24-CTCA-1	GCCACAGCCACTCACCCATTGCGT
J24-CTCA-2	ACGCAATGGGTGAGACCGAGCACG
J24-CTCA-3	CGTGCTCGGTCTCATGCAGATGCC
J24-CTCA-4	GGCATCTGCATGAGTGGCTGTGGC
J24-GTCA-1	GCCACAGCCAGTCACCCATTGCGT
J24-GTCA-2	ACGCAATGGGTGACACCGAGCACG
J24-GTCA-3	CGTGCTCGGTGTCATGCAGATGCC
J24-GTCA-4	GGCATCTGCATGACTGGCTGTGGC
J24-TTCT-1	GCCACAGCCATTCTCCCATTGCGT
J24-TTCT-2	ACGCAATGGGAGAAACCGAGCACG
J24-TTCT-3	CGTGCTCGGTTTCTTGCAGATGCC
J24-TTCT-4	GGCATCTGCAAGAATGGCTGTGGC
J24-ATCT-1	GCCACAGCCAATCTCCCATTGCGT
J24-ATCT-2	ACGCAATGGGAGATACCGAGCACG
J24-ATCT-3	CGTGCTCGGTATCTTGCAGATGCC
J24-ATCT-4	GGCATCTGCAAGATTGGCTGTGGC
J24-CTCT-1	GCCACAGCCACTCTCCCATTGCGT
J24-CTCT-2	ACGCAATGGGAGAGACCGAGCACG
J24-CTCT-3	CGTGCTCGGTCTCTTGCAGATGCC
J24-CTCT-4	GGCATCTGCAAGAGTGGCTGTGGC
J24-GTCT-1	GCCACAGCCAGTCTCCCATTGCGT
J24-GTCT-2	ACGCAATGGGAGACACCGAGCACG
J24-GTCT-3	CGTGCTCGGTGTCTTGCAGATGCC

J24-GTCT-4	GGCATCTGCAAGACTGGCTGTGGC
FAM-20dA	FAM-AAAAAAAAAAAAAAAAAAAA
FAM-20A	FAM-AAAAAAAAAAAAAAAAAAAA (RNA)
FAM-20U	FAM-UUUUUUUUUUUUUUUUUUUU (RNA)
FAM-20C	FAM-CCCCCCCCCCCCCCCCCCCCC (RNA)
FAM-20m4A	FAM-UUUUUUUUUUAAAAUUUUUU (RNA)
FAM-20m3A	FAM-UUUUUUUUUUAAAUUUUUUU (RNA)
FAM-20m2A	FAM-UUUUUUUUUUAAUUUUUUUU (RNA)
FAM-20m1A	FAM-UUUUUUUUUUAUUUUUUUUU (RNA)
FAM-20mG	FAM-UUUUUUUUUUGGGUUUUUU (RNA)
FAM-ssRNA	FAM-AUAAGAGGAGAAGGCGAG (RNA)
ssRNA-R	CUCGCCUUCUCCUCUUAU (RNA)
ssDNA-R	CTCGCCTTCTCCTCTTAT

The underlined residues in the HJs correspond to the homologous core.
