# Supplementary material for: Biochemical and Structural Study of RuvC and YqgF from Deinococcus radiodurans
Source: mBio. 2022 Aug 24;13(5):e01834-22. doi: 10.1128/mbio.01834-22 (PMC9601230; doi:10.1128/mbio.01834-22)
Supplement: FIG S6 [file mbio.01834-22-s0008.pdf]

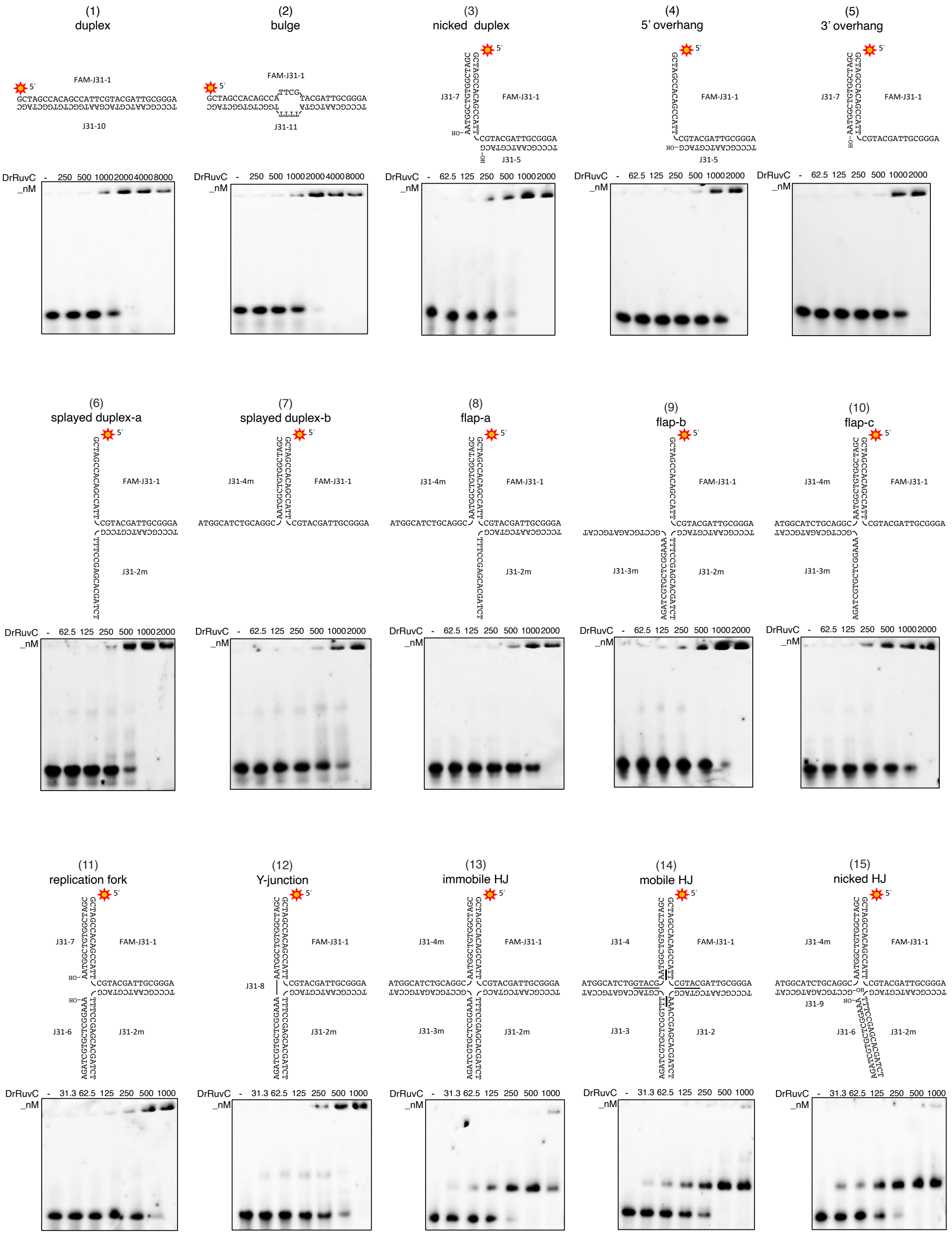

Supplementary figure S6. The EMSA assays of DrRuvC towards different DNA structures.

100 nM different substrates were incubated with different concentrations of DrRuvC (labeled on to top of each gel). The products were resolved by 5% TB-native gel.
