# Supplementary material for: Biochemical and Structural Study of RuvC and YqgF from Deinococcus radiodurans
Source: mBio. 2022 Aug 24;13(5):e01834-22. doi: 10.1128/mbio.01834-22 (PMC9601230; doi:10.1128/mbio.01834-22)
Supplement: FIG S7 [file mbio.01834-22-s0009.pdf]

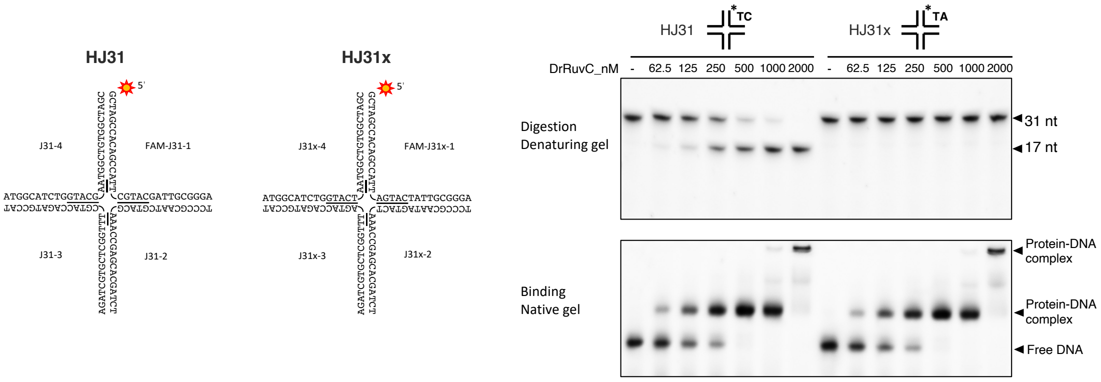

Supplementary figure S7. The digestion and binding affinity comparisons of HJ31 and HJ31x.

As for the digestion assay, 100 nM HJ31 or HJ31x was incubated with different concentrations of DrRuvC (0, 62.5, 125, 250, 500, 1000, and 2000 nM), in the presence of 10 mM Mn^{2+} at 37°C for 30 min. The products were resolved by 15% denaturing gel. As for the binding assay, 100 nM HJ31 or HJ31x was incubated with different concentrations of DrRuvC (0, 62.5, 125, 250, 500, 1000, and 2000 nM). The products were analyzed by 5% TB-native gel.
