# Supplementary material for: Biochemical and Structural Study of RuvC and YqgF from Deinococcus radiodurans
Source: mBio. 2022 Aug 24;13(5):e01834-22. doi: 10.1128/mbio.01834-22 (PMC9601230; doi:10.1128/mbio.01834-22)
Supplement: FIG S5 [file mbio.01834-22-s0007.pdf]

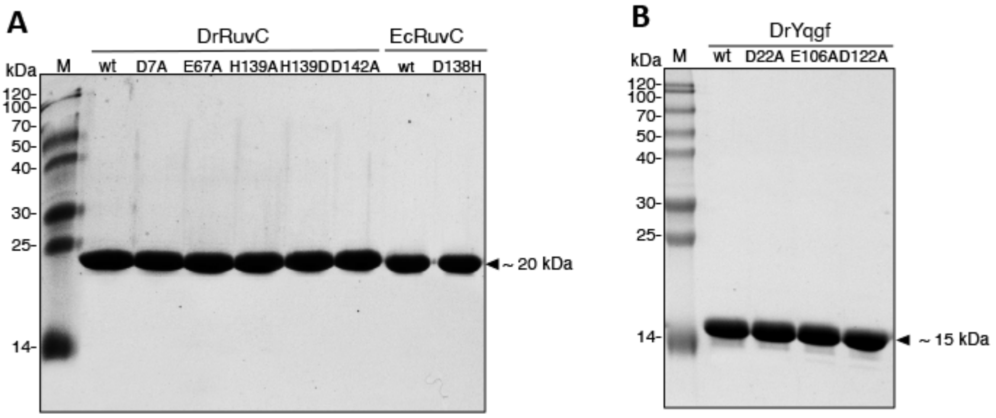


**Supplementary figure S5. Protein purity verifications.**

**(A)** The purities of purified DrRuvC and EcRuvC and related mutants were analyzed by 15% SDS-PAGE. **(B)** The purities of purified DrYqgF and related mutants were analyzed by 15% SDS-PAGE.
