# Supplementary material for: Biochemical and Structural Study of RuvC and YqgF from Deinococcus radiodurans
Source: mBio. 2022 Aug 24;13(5):e01834-22. doi: 10.1128/mbio.01834-22 (PMC9601230; doi:10.1128/mbio.01834-22)
Supplement: FIG S8 [file mbio.01834-22-s0010.pdf]

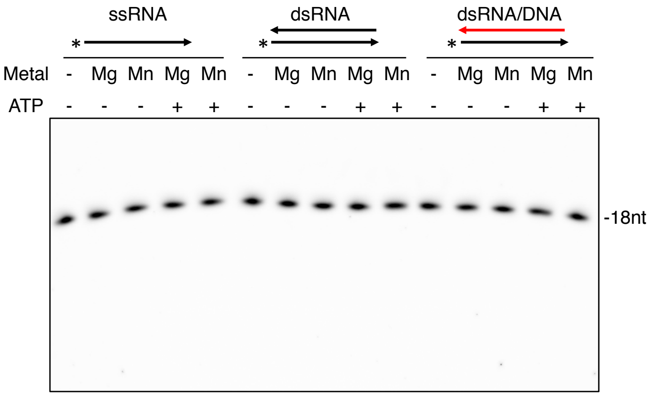

Supplementary figure S8. The RNase analysis of drYqgF on short RNA substrates containing random sequence.

200 nM ssRNA, dsRNA and dsRNA/DNA substrates with random sequence were incubated with 1 µM wild type DrYqgF and 10 mM metal (Mg^{2+} or Mn^{2+}) at 37°C for 30 min. Reactions were carried out in the absence or presence of 5 mM ATP. The products were resolved by 15% TBE-urea denaturing gel.
