# Supplementary material for: Biochemical and Structural Study of RuvC and YqgF from Deinococcus radiodurans
Source: mBio. 2022 Aug 24;13(5):e01834-22. doi: 10.1128/mbio.01834-22 (PMC9601230; doi:10.1128/mbio.01834-22)
Supplement: FIG S3 [file mbio.01834-22-s0005.pdf]

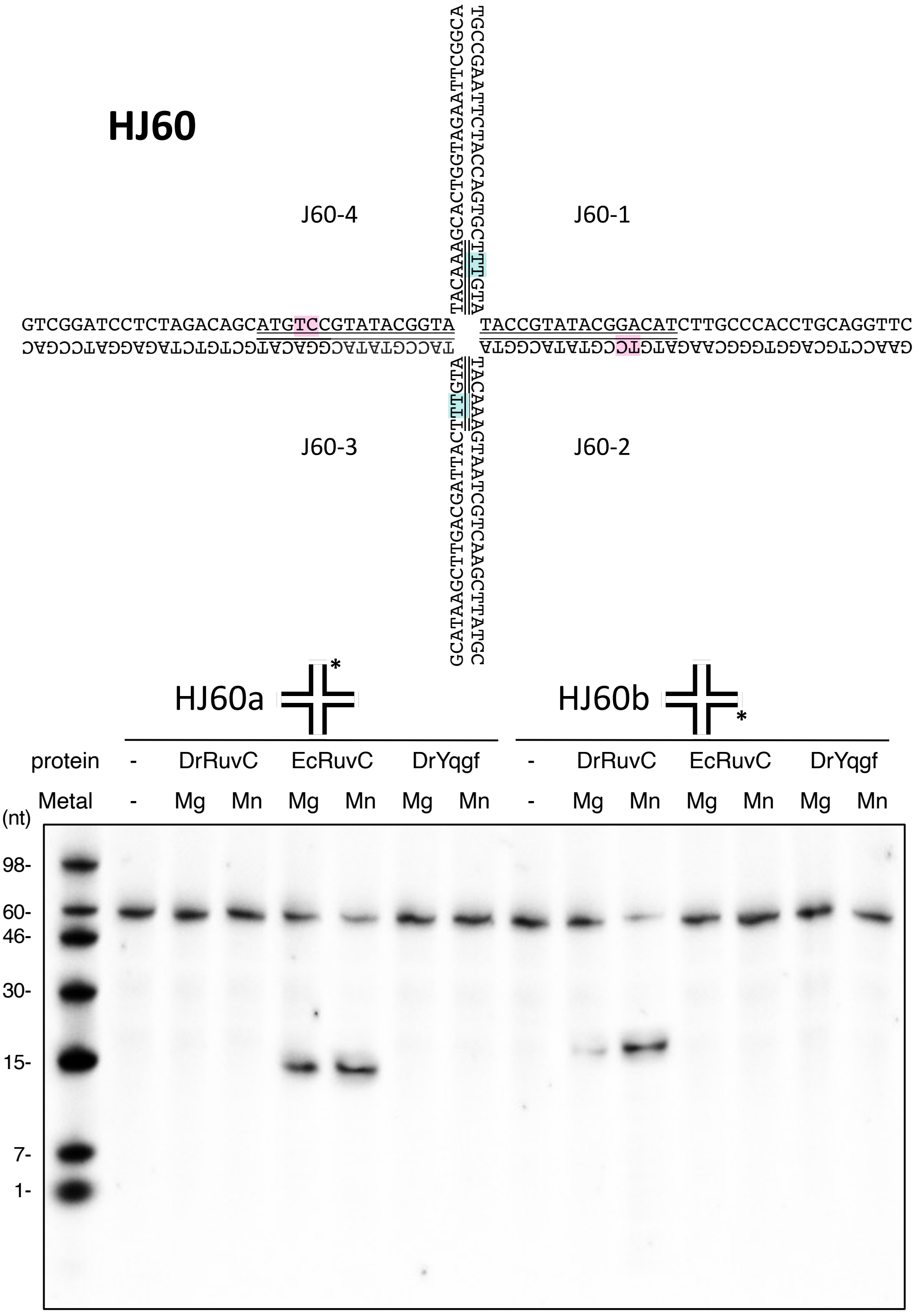

Supplementary figure S3. Analysis of the HJR activities of DrRuvC and DrYqgF on long HJ substrates (HJ60).

5’ 6-FAM labeled HJ substrates with 22 nt homologous core were synthesized (labeled at strand J60-1 or J60-2) to monitor the different cutting patterns. 200 nM DNA was mixed with 1 μM protein, 10 mM metal (Mg^{2+} or Mn^{2+}), and incubated at 37°C for 30 min. Products were resolved by 15% TBE-urea denaturing gel. EcRuvC was set as a control.
